# Emerging subdisciplines in ethnology and anthropology of Serbia: research trends at the Faculty of Philosophy, University of Belgrade

**DOI:** 10.1186/s41257-023-00082-3

**Published:** 2023-03-30

**Authors:** Vesna Vučinić Nešković

**Affiliations:** Department of Ethnology and Anthropology, Faculty of Philosophy, University of Belgrade, 18-20 Čika Ljubina Street, Belgrade, 11000 Serbia

**Keywords:** Serbia, University of Belgrade, Department of Ethnology and Anthropology, Scientific production, Subdisciplines, Novel trends

## Abstract

This article presents the in-depth analysis of the disciplinary landscape of ethnology and anthropology in Serbia within the institutional contexts of humanities and social sciences. Focusing on the Department of Ethnology and Anthropology at the Faculty of Philosophy, University of Belgrade, it provides insights into the main subdisciplines, fields, and themes of research since 2006, a time when significant publishing activity was invigorated and the Bologna Process reforms were implemented in Serbian universities. Using a theoretical approach to knowledge production as a complex mosaic of complementary research, rather than a hierarchy of different quality works, the article tracks the dynamics of disciplinary (re)orientations in the Department over the past 16 years. This is accompanied by a methodological approach whereby the author does not assume the role of an epistemic arbiter who selects and labels representative work, but instead invites members of the studied Department to exercise the selection processes by filling out a survey that the author composed and distributed. The article is based on information collected through the survey, the Department documentation, as well as the author’s own interpretation of the published works. Related subdisciplines are grouped in larger wholes and presented in counter-alphabetical order of the names given to them. Finally, the concluding part discusses the innovative and dynamic developments of the Department’s faculty research.

## Introduction: intentions and approach

The task of depicting the present state of the discipline of ethnology and anthropology within any national boundaries, including those of the Republic of Serbia, is not an easy one. It is however a provocative endeavor for someone who was certain that never in her professional life will she undertake such a task. The main reason of keeping away from writing on the contemporary history of our discipline has been that such an endeavor usually asks for choosing representative works within the large pool of professional publishing production. This means mentioning some and neglecting other authorship. Instead of looking at scientific production primarily as a hierarchy of different quality works, I rather look at scientific production as to a complex mosaic of complementary research that can altogether contribute to a better understanding of the natural and human worlds as an inseparable whole.

I made a choice to concentrate on the subdisciplinary scene as represented in the research of the Department of Ethnology and Anthropology at the Faculty of Philosophy, University of Belgrade. I decided to stay within the boundaries of the research activities of our department, whose activity I have participated in and observed since 1988. This decision was based on the fact that this is the home educational institution of our discipline since the inception of ethnology as a university study and is the only such exciting institution of the kind in Serbia so far. In addition, it is the only institution that unites research and teaching in ethnology and anthropology in the country.

The core of the article is therefore devoted to the analysis of the advancements of the University of Belgrade Department of Ethnology and Anthropology since 2006, when the significant publishing activity was invigorated with the relaunch of one and establishment of another department journal,^1^ and when the implementation of the Bologna Process reforms started at the University of Belgrade and all the other universities in Serbia. This article aims to present the subdisciplines, fields and themes of research of the Department and its members, based on the categorization and naming given by the faculty members themselves.

In order to remain with the principle of not evaluating my colleagues’ research and publications by naming myself the subdisciplines which they act within, and by choosing representative authors and their works, I decided to ask them to individually name and evaluate their own work. I thus formulated a short questionnaire, which asked them to write a summary of their overall research contribution by naming three subdisciplines to which they contributed the most. For each subdiscipline they were to mention: the fields and themes they were engaged in, the theoretical and methodological approaches they applied, the self-assessed contributions, and selected bibliography of their three most important publications.

Of the 27 faculty members, all replied except a few who were prevented due to objective circumstances. I found the missing data in the official bibliographies and committee reports written at the time of their professional advancement. While organizing the data, I first created a list of all faculty members and divided them into two groups – the senior and the junior faculty. The reason for this grouping is based on a clear distinction not only in age, but also in the period when they joined the department. The senior faculty, consisting of 15 members, was initially employed between 1980 and 2004, while the junior faculty, consisting of 12 members, were employed between 2016 and 2022. The 12 junior members of the Department work part-time as researchers at the Institute of Ethnology and Anthropology (which is within Faculty of Philosophy) and part-time as lecturers at the Department, thereby integrating ongoing research and teaching. While making a common list of the mentioned subdisciplines, in majority of cases they remained as mentioned by the respective faculty. Only in few cases, I changed them slightly in order to enable grouping of related or compatible subdisciplines or fields into a larger whole.

Before presenting the results of the analysis, the benefits and limitations of the role of the analyst of one’s own department should be mentioned. Probably the most important benefit was that I possessed inside knowledge of the Department, based on participation and observation of its life within a 34-year period. More particularly, this meant being acquainted with its organizational, academic, and research processes as well as with the genealogies of different subdisciplines that emerged in interaction of structural requirements of the curricula and research interests of individuals and smaller groups within the faculty. Another benefit came out of my good relations with all Department members, who out of collegial trust, made the effort to review their up-to-date research and summarize it for the purpose of responding to the questionnaire.

At the same time, I do not see serious limitations of my position as the analyst of my own department. The relatively uneven and loose structure of this article is less a result of my subjective preferences for certain subdisciplines, fields and themes, or authors for that matter, and more a consequence of my approach (which was based on showing innovative individual scientific contributions rather than classifying them in groups or making comparisons), the number of disciplines the individual Department members developed their interest in, the character of their publications (some primarily published articles, others books), but also of the different ways they summarized their work. In cases where new or less known subdisciplines or concepts were employed, more space was needed for their explanation.

## Emerging subdisciplines, fields and themes of research at the Department of Ethnology and Anthropology^2^

When thinking about the emerging trends in Serbian ethnology and anthropology, my aim was to present all the subdisciplines that have been developed through research and teaching at the Department. Some of the subdisciplines that developed in the previous decades have shown innovativeness in development of new fields and themes, while other subdisciplines have emerged only recently.

While making a common list of the mentioned subdisciplines, in majority of cases their names remained as mentioned by the respective faculty. In few cases only, I changed them a bit in order to enable grouping of related or compatible subdisciplines into a larger whole. In other cases, I treated them as fields of study within larger subdisciplinary frameworks.

The structure of this part of the article is organized according to these larger wholes, whereby each comprises of at least two or more related subdisciplines. These wholes (groups) are presented according to the counter alphabetical order of their names. Within each group, the subdisciplines follow the order given in their titles, and are described according to the fields and themes mentioned by the faculty engaged in their development. In this, I tried to combine the chronological principle of their development, and the order of seniority of the faculty that engaged in them. The fields and themes of each subdiscipline were given as a summary of the sum of individual engagements, with references to their publications pointing to individuals as contributors.

The titles of the larger wholes are: (1) World Cultures, Multiculturalism and Cultural Heritage, (2) Urban Condition: Life and Culture of Cities, (3) Science, Education, and Methodology, (4) Social and Cultural Diversity: Religion, Ethnicity, Migrations, Kinship and Gender, (5) Politics and Economics, (6) Material Culture and Fashion, (7) History (and Politics) of Ethnology and Anthropology, (8) Human Condition: Body, Cognition, Emotions, Old Age, Health and Wellbeing, Medicine and Pandemics, (9) Folklore and Popular Culture, and (10) Arts: Visual, Literary and Musical.

### World cultures, multiculturalism, and cultural heritage

Studies in *World Ethnology/Anthropology* originated at the Department since the early 1970s, with the introduction of the course on Ethnology of the world peoples, then divided into the Ethnology of the Old World and Ethnology of the New World, initiated by Srebrica Knežević to deal with ancient civilizations and contemporary cultures of large world regions.

This subdiscipline has in the meantime developed in few directions, or rather extended to three more specific regional studies, namely: Anthropology of Africa, Anthropology of East Asia, and Anthropology of the European Union. Each field developed in its own way, and its specific themes of inquiry.

*Anthropology of Africa* has had long presence at the Department. Following the needs of the course curriculum, her own research interests, and the possibility of spending 3 years in West Africa in mid 1980s, Senka Kovač focused on the exploration of West Africa. After first studying the contemporary and traditional aspects of material culture in Dakar (Senegal), she set on to analyze the “secret language” of the west African masks, i.e. the forms, functions, and communicational values of these important ritual objects (Kovač 1986, 1996). In the more recent period, Kovač focused on the contribution of French anthropologist Marcel Griol, who in the first half of 20^th^ c. conducted noteworthy field research among the Dogon of Mali. Concentrating on the methodological aspects of the original and the repeated researches, her publications analyze academic discussions of Griol’s work by his contemporaries, but also by later critics who made research with the Dogon at the end of the 20^th^ century (this particularly refers to van Beek’s work from 1992). The overall contribution of Kovač’s research is in pointing out the significance as well as problematic aspects of repeated research and discussions surrounding them (Kovač 2007a; Kovač and Milenković 2006).

*Anthropology of East Asia* appeared with the interest of Vesna Vučinić Nešković in the ancient civilization and modern society of China and Japan, which she pursued at Harvard University master’s program in Regional Studies – East Asia in mid-1980s. In this and the following period, she concentrated on the study of traditional and modern urban planning in China, and especially on the analysis of official debates on the use and value of urban land in China’s major cities in 1980s, which was the early period of economic reforms (Vučinić 1986, 1993). Participation in the “Design in a Historic City” studio, organized by MIT and Kyoto University in Japan in 1986, added to Vučinić’s interest in how city planning determines daily and ritual life in East Asian cities. After transposing her research to Southeast Europe (i.e. former Yugoslavia) in early 1990s, she revived her interest in East Asia in 2010s, while researching urban phenomena in China’s dynastic, Maoist, and reform periods. Two main research fields may be recognized, namely *urban culture and society of China*, and *cultural and health diplomacy within the Belt and Road Initiative*.

The themes and topics explored within the first field are social exchange and power relations in the traditional urban culture as represented in the classic novel *Jing Ping Mei* (from Ming Dynasty), as well as urbanization of Chinese countryside in the late Maoist period carried out through introduction of industrial rhythms in agricultural production processes and ideological activism in peasants’ after-work activities (Vučinić Nešković 2012a, 2010a). The author also dealt with the transformation of urban public spaces in contemporary China by analysing the dual symbolism based on oppositions of traditional–contemporary and Chinese–Western inscribed in urban planning, representative buildings, as well as in elements of cultural tradition (cuisine, drinks, Beijing opera) (Vučinić Nešković 2009). The second field concentrates on China’s relationship with the world through economic and cultural diplomacy. Special attention is placed on China’s health diplomacy during the Covid-19 pandemic and exchange of direct medical assistance with different regions and countries of the world, especially with the 16 + 1 Initiative members, and with Serbia. In addition, the author engaged in the study of urban culture of Japan, with focus on social interaction in open public spaces, which she explored during a four-month fieldwork in Osaka, Kyoto and Tokyo in 2017. This research was part of Vučinić’s interest in comparing urban lives in Southeast Europe and East Asia.

*Anthropology of multiculturalism*, a subdiscipline developed since 2000s by Miloš Milenković within World Anthropology studies at the Department, in time generated a few distinct anthropological subdisciplines. These are Anthropology of the European Union, Anthropology of international relations, and Anthropology of cultural heritage.

While initially exploring multiculturalism theoretically, later research within *Anthropology of the European Union* performed by Milenković and his younger collaborators (graduate students, who later become faculty members), focused on legal and cultural consequences of the EU accession processes awaiting Serbia.

It should be noted that the research on the Anthropology of the EU coincided with the Republic of Serbia submitting the official application for the EU membership (2009), and intensified when Serbia received the full candidate status (2012) and when the Council of EU approved opening negotiations for its succession (2013). The first investigative interest of the EU per se, critically considered the development of specific cultural politics undertaken by the EU institutions and their intention to constitute “European identity and culture”. By analyzing official EU Commission documents and discourses, Gačanović shows that the idea of “*European identity*” represented an amalgam of inconsistent ideas, based on inadequate conceptions of “identity”, “culture”, and “community” in the European institutions, politics and agendas (Gačanović 2009, preceded by master thesis 2008). A recent study makes a useful overview of three approaches to the study of the EU – from above, from within, and from below (Gačanović and Kovačević 2021).

Another strain, developed by Marija Brujić, deals with *Europeanization in Serbia at the start of 21*^*st*^*century* by analyzing sociocultural transformations in the country in the period of European integrations. Relying on sources from different disciplines, the study aimed to understand self-perceptions of Europeanisation and changes in everyday life of Serbian citizens (Brujić 2016). Starting with theories of Europeanization, the author goes on to analyze the influence of European integrations on the quality of life, esp. in the sphere of health system reforms, perceptions of halal food as the Turkish or European heritage, and question of forced Europeanisation and reproduction of colonialism in the process. The same author deals with cultural representations of European integrations of Serbia among immigrants in the EU countries, especially among the Serbian Diaspora in Austria (Brujić 2018). She also considers the challenges of integration related to Serbian Orthodox Christianity (2017). In addition, Bojan Žikić dealt with *cultural notions of the European Union among educated urbanities*, who looked forward to the end of the transitional period between the dissolution of Yugoslavia and entry into the “promised land of liberal market-oriented, participative democracy and higher standards of living”, awaiting Serbia within the EU (Žikić 2013).

Other themes that appeared were related to the current challenges and strategic choices awaiting Serbia in the process of EU enlargement. The themes included the possible “culturalization” of accession criteria (Milenković and Milenković 2013a), misconceptions and opportunities related to the preservation of national identity and cultural heritage in the process of accession, additional conditionality posed on Serbia as a barrier to EU accession (Milenković and Milenković 2013b), politics of multiculturalism and their consequences in Europe, and European cultural traditions (Kovač and Milenković 2014). In the most recent period, the Jean Monnet Module project “Anthropology of the European Union” gave rise to the teaching and research devoted to this field, implemented at the Department in the 2017-2020 period.

Overall, it may be said that Anthropology of the EU has been preoccupied with Europe as a source of cultural identity and values in normative and policy framework, as well as discussions on how anthropology may be useful in adaptation of the concept of multiculturalism to political reality in general, but also to the reality of Serbia (Gačanović 2010, 2011).

Interest in multiculturalism and international politics also gave way to development of research within the field of *Anthropology of international relations*, concentrated around the role of Yugoslavia and Serbia in the Nonaligned Movement. Marija Brujić explored foreign policies of the Socialist Federal Republic of Yugoslavia (SFRY) during the Cold War and of the present-day Republic of Serbia (Brujić 2011), as well as the Nonaligned Movement, in which Yugoslavia played one of the key roles (Brujić 2013).

Within Anthropology of multiculturalism, Miloš Milenković has himself been engaged in developing the field of *identity politics and cultural heritage*. The themes that preoccupied his attention have been: possibilities of preserving national cultural heritage within the EU, inclusive intangible cultural heritage protection (as an instrument of prevention of identity-based conflicts), lessons on intangible cultural heritage from the Western Balkans, and bureaucratization of intangible cultural heritage safeguarding in post-conflict regions.

Milenković’s main stances, which he develops in numerous articles and two monographs dealing with cultural heritage is that attention of the academic community should be refocused from the repressive consequences of multicultural policies to the peaceful consequences of international and national protection of cultural heritage (Milenković 2014). The advice to those working in the field of social and human sciences (in general and in Serbia) is to involve themselves in the processes of preserving national identity and cultural heritage through, and not against, European integration. His analysis shows how a society may be improved by pointing to the mythical basis of the fear of losing one’s identity in European integration (Milenković 2013). Milenković also deals with the polemic about UNESCO, intangible cultural heritage and the role of ethnology and anthropology and humanistic disciplines in general, which may be realized through these very concepts, whereby explaining that identity as the key term should be in their eyes (and paradoxically – by themselves) transformed from an instrument for creating conflicts into an instrument of intercultural reconciliation (Milenković 2016).

*Intangible cultural heritage*^3^ has been the field of interest of Danijel Sinani, both through performing research and taking active role in recommending elements of Serbian traditional culture for the UNESCO Representative List of the Intangible Cultural Heritage of Humanity. Within the first domain, focus was on the relationship between religion, identity and cultural heritage, whereby dealing mostly with theoretical concepts and legal regulations (Sinani 2010b, 2011). In the second domain, he crucially contributed to the inclusion of the first two Serbian cultural elements – *Slava*, the celebration of the family saint protector’s day, and *Kolo*, the traditional circular dance – in the UNESCO representative list in 2014 and 2107 respectively.

Within Anthropology of cultural heritage, which she calls Heritology, Jelena Ćuković developed interest in representation of heritage in museums and public discourse, differences in interpretations and constructions of cultural heritage (i.e. dissonance of heritage), authorized discourse of interpretation, and the use of cultural heritage in politics and tourism. Special attention was given to intangible cultural heritage. This concept, devised by UNESCO in 2003, by definition recalls the essence of the research subject of ethnology and anthropology. In this sense, the focus of her research was “representativeness” as the basic difference between two discourses – the authorized heritage discourse of UNESCO, and the discourse of the Republic of Serbia, and its Autonomous Province of Vojvodina. Ćukovic’s research shows that descriptive cultural relativism (not universalism) is doctrinally used and embedded in the policy of selecting elements for the UNESCO List of Elements of Intangible Cultural Heritage, and that intangible cultural heritage, as the core or carrier of collective ethno-confessional identities, is a socio-cultural construct that is subject to interpretive manipulation. Finally, it shows that UNESCO’s system of CH protection gave too much power to individual states to select elements of cultural heritage preceding research, protection and preservation, which also gave them the power to exclude the heritage of minorities, migrants or citizens to whom ethno-confessional identities are not of primary importance (Ćuković 2018, 2019; Ćuković and Milenković 2020).

Different themes related to cultural heritage have been of wider interest to the Department, as noted in books that appeared as collective projects. The first edited volume, *Cultural Identities as Intangible Cultural Heritage* (Žikić 2011) primarily discusses the conceptual and theoretical approaches to this topic, as well as the religious, ethnic, urban, historical, educational, migrant, and foreign identity determinants in the making of contemporary cultural identities in Serbia. The second edited volume, *Essays on Yugoslav Cultural Heritage* (Kovačević 2012) was conceived to represent the cultural products derived from the main features of the socialist state ideology (1945-1992), constituted on the ideational triangle of self-management, non-alignment and national defense, and accompanied by the ideas of brotherhood and unity, conceptualization of the working class as the subject of the Yugoslav and world revolution, the rule of the communist party, and the cult of Josip Broz Tito.

### Urban condition: life and culture of cities

Urban phenomena became points of interest of ethnologists in Serbia in mid 1980s, which became explicit at the conference of the Serbian Ethnological Society, entitled “The Urban and the Contemporary” (1984). Since this period, many of the Department members performed their research in urban areas, but with focus on varied issues which did not question the uniqueness of the urban experience. Another words, the city *became a locus*, but not always *a focus* of anthropological research. A distinction between anthropology within city limits, and *Urban anthropology* more strictly conceived, where the focus is on urbanism itself and its resulting socio-cultural processes and phenomena, remained for a while. The first complex monograph that tried to grasp the integral life of the city through the study of (socio-)spatial behavior of its inhabitants appeared in the early 1990s, as a doctoral thesis of Vučinić (1993), later turned into a book entitled *Spatial Behavior in Dubrovnik: Anthropological Study of a City with Orthogonal Structure.* This study made a connection between the morphology of the city, everyday life and public rituals (Vučinić 1999).

In the 2000s and further, the main impetus to Urban anthropology in Serbia was given by the 3rd Conference of the International Association for Southeast European Anthropology (InASEA), entitled "Urban Life and Culture in Southeastern Europe" (2005), organized by Vesna Vučinić Nešković and hosted by the Faculty of Philosophy, University of Belgrade (Vučinić Nešković and Brunnbauer 2005). Since then, three major research fields have developed within Urban anthropology at the Department, those of socio-spatial behavior, revitalization of neglected urban spaces, and acute urban crisis. *Socio-spatial behavior* was primarily studied through the informal social institution called *the corso* (urban promenade) which, as analysis showed, can be considered the total social phenomenon, as defined by Mauss (Vučinić Nešković and Miloradović 2006). The leisure stroll in the central city street or square, for the purpose of self-presentation and exchange of news within a local community, is practiced by social groups that vary according to age, gender, rural or urban origin, social status, and ethnicity. The themes of research included transformation of the corso from early 20^th^ to the beginning of 21^st^ century, relationship between the spatial and social structure of the city, and processes of social dynamics, such as social distancing and social integration in public space. Socio-spatial forms of interaction were also viewed in comparative perspective (Vučinić Nešković 2018), first considering cities of Serbia, Montenegro, and Croatia (based on ten empirical case studies), and more recently in urban settings of Southeastern Europe and East Asia. *Revitalization of neglected urban spaces* through situational theatrical activism was the second research field dealt with. Three performances that featured at the Belgrade International Theater Festival (BITEF), produced by young international artists, used abandoned courtyards in the center of Belgrade to question the reasons for their neglect by the public institutions and local residents (Vučinić Nešković 2012b). Third research field concerned *acute urban crisis* in Serbian cities, such as forced displacement of Roma people due to large infrastructure projects, sudden waves of refugees from the Middle East that found lodging in urban public spaces, management of the great flood of 2014, and transformation of daily life in the time of corona virus (Vučinić Nešković 2020).

An exemplary contribution to urban anthropology where an anthropologist and an architect joined their expertise is to be found in the study of the spatial and social neighborhood in a central Belgrade quarter. The study set on to determine types of old (court)yards and modes of neighborly relations in the part of Vračar that are steadily disappearing because of piecemeal and inadequately regulated urbanization. Research shows that the type of yard (which may be enclosed and open) does not influence the neighborhood relations, while the entirety of factors related to contemporary urban life in big cities does. One of the most important factors that alters the neighborhood’s landscape and neighborhood relations is the thrust of investment building, towards which inhabitants of Vračar yards have negative attitudes. In conclusion, the authors emphasize the necessity of collaborative engagement of anthropologists and architects. Instead of buildings which don’t appropriately relate to urban context and city structure, construction of buildings which are more aligned to principles of quality habitation is needed (Kovač and Kovač 2010). Another combined effort of the same two disciplines is present in the research on the influence of urban development on the borders and perception of Bežanija, the suburban part of Belgrade (Dražeta and Stankić 2020).

Other Department members engaged in themes related to urban anthropology, which is exemplified in an edited volume entitled *Urban Cultural Identities and Religiosity in a Contemporary Context* (Sinani 2013d). Themes in this volume related to urban issues include: general problems that may be encountered in the study of identities related to their ethnic, gender, regional, local, group or individual parameters, proposal for founding of new multidisciplinary field of “Belgrade studies” where cultural identities would be researched based on Belgrade as the cultural symbol, as well as exploring the history of aging in the city since mid-19^th^ century, which has been developing in a different way than aging in rural areas. In the domain of popular culture, the themes are related to: specificities of local conceptualizations of rock n roll and urbanity in the case of new wave music in Belgrade (appearing in 1980s and 1990s), phenomena tied to the metropolis as a place of “urban horror” in modern literature, types of post-apocalyptic future cities as represented in cinematography (namely the futuristic city of advanced technology and a small city of backward technology), as well as varied collective and individual identities formed around football fan practices.

### Science, education, and methodology

*Anthropology of science and education* is a relatively novel and diversified subdiscipline, which emerged in the late 2000s. It has been developing in few different strains, first of which is devoted to the critical study of science and education policies, the second to history of methodology, and the third to practical methodologies.

After being signed by Republic of Serbia in 2003, implementation of the Bologna Declaration (starting in 2006), beside positive, also brought about negative changes in the life of the Serbian universities. Another novelty at the university was introduction of quantification of research results in 2000, and the establishment of a point system for advancement in the university career in 2007.

In parallel, the Ministry of Science of Serbia, while introducing its own system of evaluation of scientific research (production) for researchers in institutes, since late 2000s opened doors for quantitative evaluation criteria to be slowly transferred from natural and technical sciences to the domain of social sciences and humanities (SSH), which was seen by the Department faculty engaged in academic politics as a dangerous trend. Ivan Kovačević and Miloš Milenković were thus at the forefront of the struggle for defending anthropology and other SSH in these externally imposed circumstances.

The main themes in their critical, activist writing included: destructive relationship of the Serbian state towards humanistic sciences at the start of 21st century, nonsense of using international citation indexes (Kovačević 2009a), moral/civilizational implications of the abolition of socio/humanistic sciences in Serbia through scientometric pseudoscience (Milenković 2009), sociometrics as a tool for destruction of interpretative sovereignty of the Serbian society (Kovačević and Milenković 2013), and false dilemmas of Serbian socio-humanistic sciences (Kovačević 2013).

In another strain, *anthropology of science* has been developed my Milenković at the intersection of history, theory, methodology, ethics and politics of social sciences and humanities, with the focus on *research evaluation and knowledge-to-policy transfer*. After extensive theorization, his explorations entered empirical waters, dealing with the problem of research assessment in Serbian academia. His recent monograph carries strong arguments against using uniform evaluation criteria in social sciences and humanities (SSH) and in natural sciences, technology, engineering and mathematics (STEM). The Serbian scholars were asked to contemplate and discuss four groups of questions, namely the overall status of SSH in academia, the evaluation criteria for SSH introduced in the previous two decades, the societal status and role of SSH, and the recent developments. The study shows that the currently predominant evaluative discourse frequently makes SSH scholars feel personally ashamed, their work devalued and their disciplines pronounced as inferior, while both institutional and individual autonomy is being diminished. The author especially underlines that evaluation criteria have significantly changed the very perception of what scholarship is, and how researchers behave. The study has proved, Milenković repeatedly states, that” the metric-based system has changed the very nature of scientific endeavor and cannot be considered objective but highly intrusive and even dangerous in social and cultural terms”. Encouraging superficiality, simplification and unreliability, the metric-based performance assessment of SSH scholars in Serbia has proved to be derogative and offensive, widely alienating scholars from their own research (Milenković 2020).

Within the same subdiscipline, Ivana Gačanović devoted her research to the field of *anthropology of higher education system*. Her long-term engagement has been the study of the process of transformation of academic culture (Gačanović 2019). Her work draws attention to the dynamics of changes in the institution of the university as well as to the factors that influence it. It also contributes to the comparative study of higher education systems in the world.

The themes of inquiry in her major monograph on this issue are revision culture, university reform, scientometrics, knowledge society, global university rankings, mercantilization of education, and political neoliberal context. These concepts and related processes are the key to understanding the contemporary global academic situation in the light of the so-called “university crisis”. The author then analyzes the importance of contemporary academic reforms in the University of Belgrade, as the largest Serbian academic institution. The stances of the respondents chosen among the university staff, on the development of the academic community, and the quality of studies, are here for the first time clearly articulated and explained. On the research side, their answers indicate that the policy “publish or perish” is counterproductive to the idea of stimulating the scientific and teaching staff to publish as large number of scientific articles as possible in prestigious foreign journals. Namely, there is a counter-effect that is reflected in reduced quality of scientific works, lectures and work with students. Analysis of this whole complex of “audit culture” in Serbia, resulting from implementation of the Bologna Declaration is placed into the international, primarily European but also world-wide context. Gačanović questions the claim about the measurability of quality, and points to problems such as uneven evaluation of scientific and teaching work, different methodology that leads to different ranking positions of the University, and problematic nature of indicators of quality. She emphasizes that critics of the revision in the world and in Serbia do not try to avoid public responsibility, but remind that it is possible to conduct the audit in other ways, such as through introduction of more specific criteria that are relevant for humanities and social sciences.

Beside the strain which represented the critique of scientific and educational policies, another strain of anthropology of science deals with *intellectual aspects of anthropological methodology* within a wider spectrum of historical and contemporary social and humanistic sciences. This direction has been taken by Nina Kulenović, whose three main fields of interest have been general methodology of social and humanistic sciences, methodology of ethnology and anthropology, and history of ethnological and anthropological methods. All of them have been approached through the prism of polemics about (scientific) explanation in socio-cultural anthropology. Within general methodology, the author asked the question of whether anthropology is a science or an art, skill, tool for loosening ethno-epistemological constraints, a comparative lens for looking at an alternative to one’s own way of life, or a tool for cultural criticism of one’s own society.

Within the field of methodology of ethnology and anthropology, Kulenović wrote two monographs that form a whole, and are dedicated to re-examining the sources, flows and outcomes of polemics about explanation in anthropology. The first one shows how the crisis of theories, methods and authority that anthropology faced in the 1980s is inextricably linked to the polemics about explanation in anthropology, which originate in the late 19^th^ century. It also shows that there is a strong correlation between theoretical and methodological structures in anthropology, and that the explanation, i.e., the explanatory capacity, is a point of convergence that can serve as a support for checking whether the separation of theoretical and methodological structures is possible (Kulenović 2016). The second monograph advocates the position that (instead of the normalization of anthropological methodology) value distancing from the object of one’s own study is the only available way to achieve objectivity, which is understood as the regulatory ideal of science (Kulenović 2017).

Within the field of history of anthropological theories and methods, individualist-holistic dispute about the concept of culture in anthropology is rehabilitated in the context of the Enlightenment—counter-Enlightenment dispute. Kulenović analyzes the dispute about whether anthropology is a “scientific” or “historical” discipline, showing that this dispute, which has been conducted throughout disciplinary history until today (and which revived especially in the 1960s and after the 1980s), is only a reproduction/reactualization of the disputes during the 1920s in anthropology, but even earlier, i.e. at the time of the institutionalization of the social and humanistic sciences in the 19^th^ century (Kulenović 2021).

Finally, there is a strain of anthropology of science that deals with *practical methodology*. Beside individual articles devoted to certain aspects of this process, *Methodology of Anthropological Fieldwork: From the Normative to the Experiential* (Vučinić Nešković 2013) is the first monograph in Serbia centered on discipline’s methodology. Normative guidelines and directions as to how anthropological research should be conceived, implemented, interpreted and presented to public, are complemented by personal experiences of the author from her three main research projects. While integrating all the above aspects of doing anthropology, the book focuses on the principal fieldwork methods, encompassing qualitative and quantitative approaches.

### Social and cultural diversity: religion, ethnicity, migrations, kinship, and gender

Development of modern studies of religion at the Department, initiated by Dušan Bandić in 1980s, bourgeoned into a rich subdiscipline of *Anthropology of religion*, with a variety of fields and themes. The core of anthropological production in the more recent period incorporated two major fields of inquiry, namely the traditional religion and the alternative or new religions. Even though the two fields sometimes overlap, the first primarily deals with the folk religion, which is primarily tied to Serbian Orthodox Christianity, the dominant religion in Serbia. The second field is concerned with nontraditional religious groups and alternative spiritual movements that have been introduced from abroad in various ways, but also with how they amalgamate and make hybrid new spiritual structures with the traditional religious concepts, beliefs and practices.

Relying on social constructivism, theory of rational choice and neo-functionalist theories, Lidija Radulović explored gender aspects of demonology in the folk religion of the Serbs, culture of memory and religious life in socialism, revitalization of religion in postsocialist Serbia, folk and official Orthodoxy in Serbia. She also dealt with conceptualization of family in religious education textbooks, occultism and fashionable cults in Belgrade, new forms of spiritual communication through Facebook, feminization of pilgrimages, and religious life in prisons (Radulović 2007, 2012; Radulović and Kovač 2015).

In her book *Religion Here and Now: Revitalization of Religion in Serbia*, Radulović views folk Orthodox Christianity as the actual, subjective, and internal reflection of official Orthodox Christianity, which is interpreted and lived differently in everyday life, and on the basis of which she proposed ideal-typical models of believers, namely the “authentic devoted believer”, and the “authentic non-church believer”. Her research informs on the complex parallel processes of revitalization of religion, retraditionalization, and invention of new traditions in the postsocialist Serbia (Radulović 2012).

Themes studied by Danijel Sinani while dealing with traditional folk religion included mythical creatures, the phenomenon of possession and exorcism, magic and witchcraft, folk medicine, ritual functionaries and holy places, periodic and special rituals, and restoration of communal celebrations in postsocialism (Sinani 2007, 2009a, 2013a). His major monograph, *Demons and Rituals* (Sinani 2013b), brings in a comprehensive approach to the most significant phenomena in the field of demonology. The study reveals the hidden “dogmas” of folk religiosity and presents them as a uniform system that has an important function in the organization of life – of both traditional and modern, rural and urban communities in Serbia. It is focused on the previously insufficiently established basic demonological categories of Serbian folk religion, which reflect the traditional ideas about human behavior and organization, its connections with nature, and with other human groups.

In the domain of alternative religions, Sinani studied theories of new religions as well as their theologies, mythologies and practices. He also worked on creating typology of religious communities and developing methodological *instrumentarium* in the study of new religions (Sinani 2009b, 2010a, b, 2013c). Sinani’s work incorporates links between religion, cultural identities, and cultural heritage (Sinani 2011). His approaches have been inspired by social constructivism, neo-functional theories, and communicational theory of culture.

In addition, Sinani contributed to applied themes, dealing with state policies and new religious groups. In the article on the visibility of religious organizations in Serbia, he deals with the legal regulations related to religious organizations and shows that due to inadequate solutions regarding their registration, a large number of religious communities are registered as non-governmental organizations with inexplicit goals and activities or are not registered at all.

Beside the above-mentioned authors whose main teaching and research focus has primarily been anthropology of religion, other Department members have contributed to the same subdiscipline. Vesna Vučinić Nešković’s contribution is visible in the exploration of revival of religious festivities at the meeting point between socialism and postsocialism in Croatia, Serbia, and Montenegro. Her focus has been on Christian communities -- Catholic (in Croatia) and Orthodox (in Serbia and Montenegro). Themes of her research include transformations of religious celebrations from the era of the monarchy, through socialism to postsocialism, the changing roles of the church, state and non-governmental organizations in organizing public celebrations of Orthodox Christmas, as well as comparison of celebration of Orthodox holidays in rural and urban, private and public settings. In addition, much attention was paid to exploring the connection between religion and politics, within the following topics: religious festivals as subversive activities in the struggle for national identity, public celebrations of Christmas as a “social drama” (in Turner’s terms) of national and local importance, symbolism in the community patron saint celebrations in the era of radical political changes, and the appearance of quasi-religious communities within secessional political movements in the time of disintegration of Yugoslavia (Vučinić Nešković 2008, 2012d, 2015, 2022). Revival of public religious festivities as a symbolic demarcation between political systems, i.e. socialism and postsocialism, has also been dealt by Sinani (2013a) in his research on Serbia.

*Anthropology of holidays* has been the field to which Senka Kovač made visible contribution. This research may be discerned within two fields, one is the study of the specific Serbian family feast devoted to the house saint protector, known as *Slava*, which included the contemporary forms of the ritual and traditional practices around the festive table (Kovač 2007b). Special attention was devoted to the same holiday after it was included in the UNESCO representative list of intangible cultural heritage of the humanity in 2014 (Kovač 2017). The other field is the study of new state holidays, the first being Sretenje (Statehood Day) – commemorating the outbreak of the First Serbian Uprising in 1804, which evolved into Serbian Revolution against the 450 years long Ottoman rule, and the second was The First World War Armistice Day. Implemented on a large survey sample, the first monograph studied the significance of the new holiday in the making of cultural identity of the contemporary citizens of Serbia, while the second focused on observance of the holiday at places of remembrance and on the reception of media messages (Kovač 2011, 2020).

Beside the studies of Christian traditions, which predominate among the population of Serbia and the states of former Yugoslavia, another strain/field of anthropology of religion has been developing in the Department, and this is *anthropology of Islam*. The research of Marko Pišev looked at Islam in light of cultural relativism, history of disciplinary thematization of Muslims in Serbian ethnology, as well as the relations between Islam and politics. His study demonstrates the advantages of cultural relativism as a research approach against more universalist-oriented theories. His book *Islam, Relativism and Science* reexamines established judgments about the clash of civilizations, and presents culturally specific perspectives of democracy, feminism, human rights, biopolitics and other social paradigms in the light of Islam (Pišev 2018). Moving outside the borders of Southeast Europe, his inquiry also examines Islam in anti-multicultural rhetoric of the West European politicians and anthropologists (Pišev and Milenković 2013).

Especially valuable is Pišev’s research of Islam in Sub-Saharan Africa, presented in the book *Between Fetish and Crescent Moon* (Pišev 2017). Approaching the problem from multidisciplinary perspective, anchored in historical and anthropological reconstruction of processes, and applying a comparative analysis of different sub-Saharan societies, the author shows that Islamic revival is a symptom of unsuccessful modernization. Another words, political Islam appears as a serious rival to the secular governments most often when they are unable, unwilling, or simply uninterested in symmetrically promoting the cultural program of the modern state for the benefit of all its citizens. Delegitimizing themselves by their persistent failure to ensure well-being and security for all members of society, modern nation-state institutions create vacuum fields that are gradually filled with radical ideologies founded on Islamic sources. The book covers the periods before, during and after colonization of Africa, keeping in the focus of its interest the coupling of political and cultural changes that started since the ninth century and remained uncompleted until today. A variety of colonial rules are incorporated, such as French, British and German, as well as the national and local Islamic responses to them.

Another strain of more recent research developed at the intersection where the *study of religion, temporality, and politics meet*. Mladen Stajić undertook comparative study of social construction of temporality through the investigation of prophetic myths in Serbia and other parts of the world. In the study on cultural construction of prophecies and precognition, the author deals with the political use of prophecies in three cultural and historical contexts - South Africa, Indonesia and Serbia. The deconstruction of three case studies shows that seemingly diverse narratives have a similar or identical basic structure, which provides insight into universal patterns of the use of prophetic discourses in promoting political ideas, goals, and individuals. Contrary to popular opinion, prophecies do not have the primary function of predicting the future, but of legitimizing the present and reinterpreting the past in accordance with the dominant political ideology. The connection between prophetic narratives and times of crisis is also discussed. Stajić shows that they can serve as catalysts for social change or represent a neutralizing factor that tends to preserve the existing order and homeostasis (Stajić 2014, 2015, 2020).

*Anthropology of ethnicity*, of which foundations were laid in 1960s by Petar Vlahović, and in 1980s by Milica Živanović and Edit Petrović, is mostly represented in the work of Saša Nedeljković. More generally viewed, the author’s interest lies in the domain of ethnicity, nationalism and distribution of power. His approach relied on ethnic identity as a relational category, empirically observed mostly through its subjective dimensions. The research encompassed two main fields, namely *identities of ethnic minorities in Serbia and Romania*, and *national identity of Serbs living in Serbia and in the USA*.

Within the first field, explored through qualitative fieldwork methods, the focus was on various aspects of identities of Montenegrins, Egyptians and Vlachs in Serbia, as well as of the Karasevs in Romania. More general themes explored within the first field dealt with an overview of ethnicity studies in Serbia, interplay of ethnicity, religion and nationalism, the role of language in ethnic differentiation, as well as with struggle for power in interethnic relations. Studies related to Montenegrins in this field encompass themes such as: theories of ethnic origin of Montenegrins, individual migrations of Montenegrins to Serbian cities in the post-WW2 period, masculinity as an alternative parameter of ethnic identity, as well as memory and identity strategies of Montenegrins in Serbian autonomous province of Vojvodina (e.g. Nedeljković 2013a). Egyptians, who have started to claim separate ethnic identity from the Roma (Gypsies) of Southeast Europe since the 1980s, have been studied through their activism of seeking right to self-declaration initiated by a complex political situation in Serbia’s autonomous province of Kosovo and Metohija (Nedeljković 2006). The author also dealt with the modern construction of identity of Karasevs minority in Romania in light of interethnic relations in Southeast Europe (Nedeljković 2017). Other important studies dealt with myth, religion and national identity in the time of national crisis in Serbia (occurring during the 1999 NATO war against Federal Republic of Yugoslavia) as well as the strategies of ethnic minority groups for climbing the social ladder (Nedeljković 2007, 2015).

The second field relied on analysis of narratives, using social psychological scales of national attachment, authoritarianism, and ethnic distance. The expressions of national identity among the Serbs have been related to national and religious identities of students in post-socialist Serbia, and more particularly of the participants in the Students’ Protest of 1996/97 (Nedeljković 2001). The research interested in the Serbian immigrants in the USA is represented in two themes. One dealt with the “economic viability of ethnicity”, i.e. the economic behavior as an expression of ethnic identity among Serbian Diaspora in the USA. The other, which included reflective self-observation approach, applied by the author himself (with insights coming from the position of a temporary immigrant), was the study of distribution and reproduction of power within the international scholars’ exchange program in the USA (Nedeljković 2011, 2013b).

Another strain of research within Anthropology of ethnicity has been pursued only recently by Bogdan Dražeta. The fields of his interest are the *construction of ethnic, religious, regional and local identities* as well as the *relationship between the normative and subjectively perceived ethnic borders*. Ethnic identification is explored alongside other kinds of identifications among the residents of two cities in Bosnia and Herzegovina, namely the Bosniaks, Serbs, Croatians and those who do not identify with any of the previous three groups. Research explores links between the post-1990s civil war administrative (formal) and symbolic (informal) borders in the cities of Sarajevo and Mostar and different levels of ethnic identification of the four mentioned groups.

Following the initial ideas of anthropology of borders, set by Barth and developed by Wilson, this work proved that ethnic identity in Bosnia and Herzegovina is maintained precisely through ethnic borders, i.e., that borders direct social life, are relational, and depend on the specific nature of interaction between two or more communities. Moreover, it showed that in the context of borders, cultural constructions of everyday life play a significant role, i.e., they strongly influence how people think and what they do in a specific space and time, as opposed to what is prescribed by institutions and the media. Based on two case studies, the research contributes to the understanding of post-conflict ethnic relations in the context of changed socio-demographic conditions and divisions of urban space (Dražeta 2019, 2021).

Other authors dealt with themes related to other ethnic groups represented in Serbia, most of all with the Roma (Gypsies). The Roma drew much attention of researchers since they are the only ethnic group in Serbia and the Balkans that are racially distinct. Most of the authors dealt with different aspects of their “culture of poverty”, displacement due to infrastructure projects, treatment by the state institutions and NGOs, or strategies of their further inclusion (Jarić and Milenković 2014; Vučinić Nešković 2020). Another theme related to the Roma people in Serbia was the construction of their minority identity through hip hop music (Banić Grubišić 2013).

Studies of migrations in and around the Balkans have had a long tradition in Serbian ethnology.^4^ More recently, *Anthropology of migrations* has been initiated in the Department within the project “‘Stranger Here, Stranger There’ – Cultural Heritage and Identity of the Migrant/Guest Workers Population” (2011-2013), coordinated by Dragana Antonijević, and supported by the Ministry of Culture of Republic of Serbia.

The project centered around *migrations from Serbia to Western European countries, especially considering the guest workers, known as Gastarbeiter*. The intention of the project was to determine the conceptual content of cultural identity of the Gastarbeiter through anthropological research, primarily based on qualitative analysis of interviews with the guest workers, their children, and compatriots who stayed in the homeland. The Gastarbeiter were economic migrants who in the early 1960s went from poor countries of southern and southeastern Europe to western European countries in search of better wages and standard of living. At the time, former Yugoslavia was the only “communist country”, which first unwillingly, and later massively and chaotically allowed its unskilled and low-skilled workers to go to western “capitalist Europe” for “private employment” (Antonijević 2013).

While applying micro and meso theoretical approaches to migrations and formal, functional, comparative, interpretative and cognitive analysis of fieldwork data, Antonijević and her junior collaborators collected most data in eastern Serbia, that is in the villages of the migrants’ origin, as well as in Vienna (Austria), the city with the most numerous Serbian Diaspora in Europe, where they met with representatives of the Serb cultural associations.

Fields and themes explored included: experiences of migrants of different waves of migrations (starting from 1955 to 2000s), life stories telling of degrees of acculturation in different countries (Austria, Sweden, Germany and France), cultural identities of first- and second-generation migrants, their attitudes towards cultural heritage from Serbia, levels of integration into foreign culture and society, deliberations on returning to homeland, survival strategies of migrants in retirement, and Serbian migrants’ associations. Overall, the research elaborates the complex corpus of shared guest workers’ experiences, which are characterized by liminality, i.e., socio-territorial bipolarity, which reflects their condition and feeling of nonbelonging to either the country of origin or the country of reception (Antonijević 2011, 2013; Antonijević, Banić Grubišić, Krstić 2011; Antonijević, Banić Grubišić and Rašić 2021; Antonijević and Milosavljević 2016). Other research within and in the follow up of the same project considers the relationship between art and migratory experiences – expressed in novels, poetry, drawings, theatre, etc. (Banić Grubišić 2018, 2019, 2020; Antonijević 2020).

Another strain of studies of migrations, this time in opposite direction, was taken on by Marija Brujić (Krstić), and these are *voluntary migrations to Serbia*. This is a unique theme since the migrations from highly developed to lower developed countries have not been subject of anthropological inquiry. Moreover, Serbia does not represent an “attractive area for employment of foreigners” except for Chinese economic migrants (Antonijević, Krstić, Banić 2013). The research focuses on highly educated female transmigrants living in Belgrade, and develops a thesis that their “transcultural capital” could have become a trigger of “Europeanisation from below” and development of the local communities, but concludes that this developmental potential in Serbia remains unused (Brujić 2021).

*Anthropology of kinship* is a traditional field of disciplinary inquiry, initially developed within the subdiscipline of Ethnology of social organization. This subdiscipline was developed by Nikola Pavković since the early 1960s, through research of traditional family, kinship, tribal and village institutions from the perspective of Legal ethnology. This field gained continuity through research of Zorica Ivanović (2008). The fields of her exploration have been within theories of kinship, ties between kinship and economy, as well as interrelationships between kinship, gender and religion. Her research reexamined classical anthropological theories related to the nature of some important social institutions in traditional societies. One such study was devoted to critique of structuralist and other Western theories related to “marriage by wife purchase” around the world (Ivanović 2007). A more recent study, coauthored with Slobodan Naumović, deals with ideological and political implications of scientific theories on formation, functioning and social consequence of the traditional Balkan *zadruga*. The family form that became known as “zadruga” (cooperative) represents a socio-economic group of two to three generations of patrilineal kin (i.e. father and his married sons), based on common life, property, production and consumption (Naumović and Ivanović 2018).

Within the *Anthropology of gender*, the Department members have been dealing with different issues this subdiscipline entails. Ivanović has been exploring a few different fields in this area, namely: transformations of anthropological theory and practice related to gender issues, relationship between feminist theory and anthropology, influence of neoliberal transformations on conceptualization of gender, structural and gender-based violence, and homosexual identities and their religious expressions (Ivanović 2007; Ivanović and Radulović 2014; Ivanović and Bobičić 2020).

Lidija Radulović developed her own research on gender along some similar paths as Ivanović, however within some distinct fields. Her early work within the field of gender and religion was on the construction of gender in the Serbian folk religion, while later it incorporated the study of gender aspects of pilgrimages, the treatment of gender in alternative religious cults, and most recently on religious and spiritual expressions of the LGBT population in contemporary Serbia. In the field of gender-based violence, Radulović analyzed narratives of women who killed violent partners, and cultural models of verbal violence against women in the public sphere (Radulović 2009, 2019; Erdei and Radulović 2020). Altogether, her studies developed into a new subdiscipline which she named Anthropology of inequality.

The field of reproductive politics has also been taken on by Nevena Milanović, whose work looks at the complexity of interrelationships between the religious (Serbian Orthodox), feminist, and folk discourses on reproductive rights and policies in the context of post-2000 political transition and secularization of Serbian society. The study critically examines crucial social institutions that reproduce “social reality”, namely: family, civil society, church, and media (Milanović 2014).

### Politics and economics

*Political anthropology* has been a subdiscipline developed at the Department since mid-1990s primarily by Slobodan Naumović, and later, since mid-2000 by Vladimir Ribić, whereby other members, namely Dragana Antonijević and Ivan Kovacević, have also dealt with it through specific strains coming from Anthropology of folklore and popular culture.

Research by Naumović incorporates three distinct fields, namely instrumentalization of tradition in political life, processes of democratization and new social movements, and Europeanization of agriculture in Serbia.

Themes of the first field – *instrumentalization of tradition in political life* -- may be distinguished as belonging to two thematic groups, one based on analysis of narratives and other of rituals. The first assumed analysis of the use of various elements of national tradition in political communication and public life during the first phase of the political transition in Serbia (1980-2000s) (Naumović 2009a),^5^ as well as of urban folk narratives about cultural, ideological, social and identity divisions in Serbia (at the turn of the 21^st^ century) (Naumović 2009b). These studies analyzed social foundations of political ideologies and political communication, offered a novel systematization of elements of national tradition, made a distinction between conservatism and traditionalism, and systematized forms of traditionalism in contemporary political life of Serbia. The second thematic group deals with the changes in the logic of reciprocity principles in village rituals (such as wedding gifts) during the economic transition, and with the use of tradition in village rituals during the political transition in Serbia (Naumović 2013a, b).

The second field of Naumovic’s inquiry within political anthropology encompassed analysis of the *role of new social movements (Otpor!) in the processes of political transition and democratization in Serbia*. This research was inspired by theories of new social movements and theory of electoral revolution. Two major studies reveal strategies of action and political role of Otpor! (Resistance) in the electoral revolution in Serbia. The most important issue addressed concerns providing adequate theoretical explanations of the “revolution of October 5^th^ 2000” in Serbia, and of the roles that the student/popular movement Otpor! played in it (Naumović 2006, 2009c).

The third field of inquiry of the same author deals with *Europeanization of Serbian agriculture* within the framework of anthropology of policy and state administration as well as theories of neoliberal economics and state. The predominant themes in Naumović's major monograph, *Fields of Paradox: Three Case Studies on the Europeization of Agriculture in Serbia* are paradoxical consequences of the managerial and entrepreneurial approach in the development of organic production in Serbia, the logic of the functioning of the first project of institutional twinning in the Ministry of Agriculture of Serbia, institutional and administrative cultures in Serbia, the process of renting agricultural land in state ownership in the context of neoliberalization of the economy, reform of the social security system and corrupt functioning of the local state (Naumović 2013c).

Other fields and themes of political anthropology have been pursued by Vladimir Ribić. The key concepts that mark his explorations are: political cultures, nationalism, political mobilization and instrumentalization, and world system theory. The author applied these concepts in the *study of different periods of Serbian political history, including its place in world politics*. The author first dealt extensively with the Serbian revolution within the modern world system, i.e. the First and Second Uprisings against the Ottoman Empire in the early 19^th^ century (Ribić 2015a). His studies also went on to examine the integralist and anti-imperialist Serbian nationalism and South Slavic pan-nationalism, that was realized owing to the destruction of the Ottoman and Austro-Hungarian empires, and the present-day European Union dislikes of these historical political movements that reached their peak at the end of WW1 (Ribić 2015b).

Special attention was also given to the breakup of Yugoslavia in 1990s, where he studied political mobilization of Montenegrin communists in “Antibureaucratic Revolution” and of the Serbian opposition in March 1991 protests, as well as nationalisms in Croatia, Montenegro, and the Serbian Autonomous Province of Kosovo and Metohija (Ribić 2012a, 2008a, b, 2019). Other themes incorporate the study of *Serbian political idiom and the prospects of European integration*. Ribić showed how after WW2, the political idiom of European integration became incompatible with the Serbian political idiom, because the idiom of European integration in the process accepted the imperial idiom based on the expansion of the American imperial mission (Ribić 2012b). Finally, his work evaluated the up-to-date Serbian political anthropology and its prospects in the 21^st^ century (Ribić 2012c).

As a somewhat distinct field, Ribić critically analyzed *Anglo-American use of applied anthropology* in Indian reservations in the USA, in the development of former colonies, and the role of American anthropologists in war (Ribić 2007a, b).

Studies of economic relations and phenomena from anthropological perspective have started to proliferate in Serbia in the early 2000s, and have thus become a foundation for further development of *Economic anthropology*.^6^ At the beginning, they were mainly concentrated on the *changes in economy through the prism of contacts between capitalist “West” and postsocialist “East”*. Research activities were grouped around two major international projects, through which the changing economic and social landscapes were mapped. The aim of the research was also to collect data for creation of applicative, research informed, policies. The pilot project later evolved into “Project Dioskuri: Eastern Enlargement – Western Enlargement: Cultural Encounters in the European Economy and Society after the Accession” (2004-2008). Both projects were designed to explore – in comparative perspective of several countries of Central-East Europe and South-East Europe – how “packages” containing liberal economic and social policies were unfolded, interpreted and employed in different national/local communities. Several case studies came out as a result of this research. Themes and topics explored by the anthropologists in the Serbian research team of the second project, coordinated by Vesna Vučinić Nešković, were: implementation of Western organizational and business culture patterns in establishment of repatriate businesses in Serbia (Vučinić Nešković 2010b, 2011, 2012c), administration of the EU Twinning project as an example of Europeanization of agricultural sector in Serbia (Naumović 2013c), privatization of a local Serbian brewery and comparison to a similar case in Slovakia (Erdei 2011; Erdei and Mareš 2012), as well as the privatization of a major mineral water and soft drinks company as reflected in media narratives (Miokov and Vučinić Nešković 2011). Recently a study of relationship between national culture and organizational culture in a multinational company office in Serbia contributed to the same field (Dražeta 2017; Dražeta and Dražeta 2020).

Beside the abovementioned studies, a thread of research on socio-cultural dimensions of *consumption* (Erdei 2008, 2012, 2018) and *cultural conceptualizations in entrepreneurship* (Erdei 2005, 2009) can be discerned. Ildiko Erdei’s monograph *Waiting for Ikea* (2012), exemplifies the complex socio-economic and cultural ambiances of Yugoslav socialism, and Serbia’s postsocialism. The first part deals with the “consumption turn” related to the image of “Tito’s pioneers” and the concept of “happy socialist childhood”. The second part considers the “local life of global icons” at the start of postsocialism, such as Vajfert brewery in the town of Pančevo, the monument of Hollywood hero “Rocky” in a Serbian village, and the public debates around the arrival of Ikea, the Scandinavian furniture company. Contribution to the subfield of economic anthropology was also made by studies of economic behavior and consumption patterns of working migrants, Gastarbeiter (Antonijević, Banić Grubišić, Krstić 2011; Antonijević 2013).

In one particular strand of writings, economic phenomena are approached through their *contextualization in urban legends and folklore*. Authors spotted new urban legends on “winners of transition”, in which motives of “unexpected gain”, previously connected with “rich uncle from America” or lottery wins, are now replaced by narratives of fortunes gained in sport betting, activity that was mushrooming in the period of “second transition” after 2000. In contemporary urban legends success became more easily measurable, materialized, and could be counted (Kovačević 2007, 2011). Personal stories of material success or failure were analyzed either as part of family folklore and idioculture of small groups (Antonijević 2009), or through their incorporation into media and popular cultural narratives (Kovačević 2007).

Finally, *informal economic life (*i.e. *alternative financial transactions) in earlier periods of Yugoslav and Serbian history* has also been the field of inquiry. Such themes incorporate the studies of financial criminal and nationalism within state financial institutions, black market transactions in Serbia during WW2 related to exchange of commodities as well as dealings with foreign currency inherited from close relatives from abroad (Nedeljković 2014). Early Yugoslav socialism was also the field of inquiry, which focused on *mechanisms through which the socialist economic system created shortages* as well as on *the complex interrelationship among the producers, distribution of goods and consumers* (Velimirović 2016, 2021a). Based on press analysis, these studies revealed systemic anomalies in the process of production and distribution of clothing. Application of Bordieu’s concept of cultural mediation, showed that continual recategorizations of the commodity values were the result of mediation of different actors on the market.

### Material culture and fashion

*Anthropology of material culture* is a subdiscipline that has emerged from a long-time interest of Serbian early ethnologists, such as Jovan Cvijić, Jovan Edeljanović, Tihomir Djordjević and others, in types of traditional rural and urban settlements, architecture, interior house design, cooking and serving ware, food and drink products, dress and accompanying decorations, tools for various activities, such as agriculture, and all the other objects that served traditional every-day and ritual life. This approach assumed descriptions of the forms and uses of material culture elements relying on the concept of regional cultural-geographic zones.

New themes started to appear since 1970s, when Djurdjica Petrović introduced a course on material culture of Yugoslav peoples, preceded with fieldwork studies of traditional weapons and their production in the Balkans. Her research continued with exploration of historical material culture in the urban context of the large trading centers such as Dubrovnik, Budapest, and Venice, which influenced the production and exchange of goods with other Balkan cities. In 1980s Mirjana Prošić Dvornić studied urban dressing in late 19th and early 20th century. Since the 2000s, new analytical and interpretative approaches were introduced in the study of material culture of Serbia. Recently, research within this subdiscipline at the Department has developed in three major fields, namely in the study of traditional material culture of Serbia, the study of socialist fashion in Yugoslavia/Serbia, and exploration of postsocialist phenomena related to material culture.

In her studies of *traditional material culture*, Danijela Velimirović investigated the influence of the political, social and economic circumstances in which the Serbs lived (Habsburg Monarchy/Austro-Hungarian Monarchy, Principality/Kingdom of Serbia, Ottoman Empire) on traditional material culture, such as architecture, food and clothing (Velimirović 2021b). She identified key political and economic turning points, which influenced the change of cultural patterns, i.e. the processes of Orientalizing, “nationalization” and Europeanization of material phenomena in the 19^th^ and early 20^th^ centuries.

Starting from Daniel Miller’s concept of objectification, Velimirović interprets artifacts as those that constitute class, gender, ethnic and national identity, neighborhood relations and privacy, the collective and the individual. Revising the culinary triangle of Claude Lévi-Strauss and adopting the theory of “doing gender” by Candace West and Don Zimmerman, the author also analyzes various embodied practices associated with elements of material culture. She uses materials from various sources, primarily those recorded in ethnographic anthologies, travel diaries, memoirs of renowned Serbian intellectuals and scientists, literary works, articles published in magazines and newspapers, as well as visual materials.

Within the *Anthropology of fashion*, Velimirović primarily deals with fashion in Yugoslav socialism. She began research in this domain by studying the designs of Aleksandar Joksimović, one of the most renown Yugoslav fashion designers of the time. Her interest continued with “socialist chic”, where she studied links between fashion, ideology and consumer culture in Socialist Republic of Serbia (Velimirović 2008). The subject of her interest was also the clothing of Jovanka Broz, the wife of the lifelong Yugoslav president Josip Broz Tito (Velimirović 2006). She is particularly interested in the relationship between femininity and fashion, and the techniques and practices through which Yugoslavia created authenticity in relation to Western fashion, but also the fashion of other socialist countries (Velimirović 2014).

Relying on Althusser’s concept of ideology as “living experience” and his concept of “ideological state apparatus”, Velimirović views socialist fashion as clothing, the continuous change of which is subject to review, valorization, authentication and discipline by central authorities. Starting from the thesis of Mary Douglas and Baron Isherwood about the social and cultural use of goods, through which initially neutral goods acquire meaning, the author understands clothing as a means of identification within the same social group, as well as a means of differentiation between different social groups. In addition, in her approach she tries to unify previously separated fields – fashion, femininity and consumption, and analyzes the interconnected discourses of ideology, politics, culture, economy, consumption, fashion, taste and gender. Her research on Yugoslav fashion is primarily based on the analysis of domestic daily and periodical press.

### History (and politics) of ethnology and anthropology

The *History (and politics) of ethnology and anthropology*, has been a long-standing subdiscipline at the Department. After the piecemeal studies, devoted mainly to renown ethnologists and in celebration of Department anniversaries, the major synthesis in this field has been written by Ivan Kovačević in two volumes of *History of Serbian Ethnology* (Kovačević 2001a), based on his doctoral thesis, *Ethnology in Serbian Enlightenment* (Kovačević 1981). This study incorporates the period from the end of 18th to the end of 20^th^ century. It analyzes the influence of Enlightenment and Romanticism on the constitution of Serian ethnology. Following the same interest, Kovačević produced new studies, recently published in *History of Serbian Anthropology* (Kovačević 2015). This is a collection of author’s articles written with the aim to *present developments in modernization of the discipline since mid-1970s* (and especially since 1990s), *with the focus on its transformation from “ethnology” to “anthropology”*. Among the themes are: the debate with Serbian sociologists (and social anthropologists) on conceptual differences between science of “people” and anthropology of “Man”, the significance of application of structuralized Van Gennep’s rites of passage model (for decoding ritual structures) in Serbia’s folklore and ethnographic material, the impact of structuralism on anthropologization of Serbian ethnology, and the role of Dušan Bandić in modernization of religion studies. Other themes of this collection incorporated: the significance of studying contemporary (socialist) rituals (e.g. Worker’s Day, Women’s Day, admission to the pioneers’ organization, enrolling for military service) for the constitution of new Serbian anthropology, revival and establishment of new anthropological journals in Serbia (2000-2010) and criticism of idolatry of journals from the SCI, SSCI, AHCI lists, and finally, the features of Serbian anthropology in the first decade of 21^st^ century.

History of ethnology and anthropology in Serbia has also been the sphere of interest of Slobodan Naumović. While examining the period of *constitution of the discipline in 19*^*th*^*and early 20*^*th*^*century*, he was interested in *philosophy and epistemology of social sciences and critique of ideological bases of scientific directions and orientations*. The themes of his exploration were: Enlightenment and Romanticism as ideological basis of social sciences, system of national sciences, educational and academic system as a function of the political goals of the national state, history of social sciences and relation to scientific heritage, and politicization and ideologization of scientific heritage (Naumović 2015, 2016). In examining the period of socialism (from mid to late 20^th^ c.), Naumović dealt with the place and role of ethnology in the time of socialist Yugoslavia and Serbia, as well as in Bulgaria and Romania. He discussed marginalization of science, its deideologization and reideologization, the invention of alternatives to national sciences during socialism, and the relationship between national communism and social sciences (Mihӑilescu, Iliev, Naumović 2008; Naumović 2008). Lastly, he uncovered the practice of implicit ideologizing of recent scientific discourses on the origin, functioning and legacy of the family cooperative, the *zadruga* (Naumović and Ivanović 2018).

Choosing historical-biographical approach, Gordana Gorunović explored in depth two fields within history and theory of ethnology and anthropology. The first field dealt with *exploration of evolutionism and historical materialism in Serbian and Yugoslav ethnology*. The study critically examined the overall contribution of Spiro Kulišić, at a time influential domestic ethnologist. The research deals with the emergence and exemplary application of the evolutionary-Marxist approach. His work proved to be an example of stagnation of theoretical-methodological aspects in the discipline during the period of the Yugoslav socialist system (Gorunović 2007). Engagement with *interpretative anthropology in American cultural anthropology* was the second field of author’s exploration. Gorunović devoted two monographs to analysis of the theoretical and methodological contribution of Clifford Geertz, including critiques and polemics his work induced in Western cultural anthropology (Gorunović 2010, 2011). These monographs and other published articles (e.g. on Claude Lévi-Strauss, Lewis Morgan, Paul Radin, Robert Murphy) have contributed to deeper understanding of history of Serbian and Yugoslav ethnology and Western socio-cultural anthropology through contextual interpretation of biographies and scientific work of some of the leading representatives or founders of theoretical-methodological paradigms.

While generally interested *in the debates on the status and relevance of postmodern anthropology, revitalized at the turn of the millennium*, Milenković produced a three-monograph series. In the first, he focuses on debates dealing with the scopes of postmodern ethnography theory by analyzing basic contexts of its origin, application and implications for modern research, teaching, as well as application of anthropology and its status on the interdisciplinary scene (Milenković 2007a). The second monograph demonstrates how anthropology has already changed its objects, theories and methods conforming itself to interdisciplinary and multicultural politics of knowledge. The main argument here is that reflexivity in anthropology can be viewed as a coordinative definition that helped anthropology survive its three crises – the crisis of ethnographic representation, the crisis of scientific realism, and the crisis of anthropological authority (Milenković 2007b).

In the third volume, Milenković introduces intertemporal heterarchy, an original model for writing the history of anthropology, which combines epistemological analyses with that of anthropology of anthropology. Intertemporality of the postmodern condition allows for a freedom in the selection of styles, once they are divorced from their chronological contexts. In the context of methodology, this serves to dispel the ties between the subject, theory, method, paradigms, institutions and historical periods. Heterarchy is borrowed from discourse analysis, in order to introduce the possibility of narrative transgression of levels. This is a radical narrative approach which introduces antihierarchical relationships between different sorts of narratives, ultimately dispelling the border between reality and illusion. According to the author, introduction of intertemporality and heterarchy to the history of anthropological methodology allows us to claim that postmodern theory of ethnography, through a reversible process of re-signification and re-interpretation, determined the notion of anthropology in the social transformation of the roles of research and education. Finally, he offers an argument for preserving the postmodern anthropology suggestions on how to solve the problem of the scientific status of the discipline, with the disciplinary core open to various interdisciplinary and intra-disciplinary concerns, allowing us to bricolage the history of anthropology regardless of paradigms, research programs, agendas or any other common denominator of various disciplinary traditions (Milenković 2010).

### Human condition: body, cognition, emotions, old age, health and wellbeing, medicine and pandemics

Studies of different aspects of the human condition, have most often been considered as separate subdisciplines of anthropology, namely as Anthropology of the body, Anthropology of cognition, Anthropology of emotions, Anthropology of old age, Anthropology of health and wellbeing, and Anthropology of medicine and pandemics. In this text they are treated integrally, and thus presented together. Research of the Department members in this domain has been developing in varied directions and with different views regarding the relationship between the mentioned subdisciplines.

While thinking through and exploring the use of body in culture, and rejecting ontological dualism between body and mind, Bojan Žikić regards nonverbal communication, cognitive anthropology and medical anthropology not as separate anthropological subdisciplines, but merely as different fields within *Anthropology of the body*.

The author’s overall theoretical framework within Anthropology of the body, has been developed from his own interpretation of Leach’s idea of culture as a communication system, from anthropological structuralism, Chordas’s notions of anthropological phenomenology, and finally from social/cultural ontology. The major fields of his research primarily relate to cultural ideas about the body, cultural use of human biophysical capacities, and physical cultural interventions on the body. The contribution of this research is in the systematization of local cultural and intercultural ideas about the body and ways of using it (Žikić 2016a, 2018).

The *cognitive anthropological* approach used by Žikić includes ideas of culture as an ideational system in the minds of community members (systematized by Dandrade), Sperber’s “epidemic of (cultural) representations” concept, and the theory of cultural development of the mind within the broader theory of cognitive niches (Žikić 2012, 2013). His research in medical anthropology has mostly relied on Rhodes’ theory of risk environment and risk behavior, with focus on socio-epidemiological themes, studying AIDS and sex work in Serbia (Žikić 2006, 2008). Finally, in case of studying the Covid-19 epidemic in Serbia, the previous two approaches were combined. The themes tackled were the individual strategies of risk avoidance and risk management, and refusal of vaccination against the Covid-19 as a case of social solipsism (selfishness), both focused on Serbia (Žikić et al. 2020; Žikić 2022).

One strain of Žikić’s interest in human condition emerged recently in a subdiscipline that he called *Misanthropology*. The concept was coined by Gary Saul Morison, one of the most respected American experts on classic Russian literature, and above all on Dostoyevsky. Morison designated this study as “the science of human cussedness”, with “cussedness” generally referring to misdeeds and disobedience, and more specifically to meanspirited disagreeable contrariness of human nature. In Žikić’s conceptualization, Misanthropology is a subdiscipline devoted to phenomena found in contemporary societies and their cultures that oppose the humanistic principles. The aim of misanthropological research is not to deal with social pathology. Instead, it is to uncover and analyze non-compliance with certain normative, publicly proclaimed values or ideals. Besides developing this field in terms of combining anthropological theories of the body and theories of cognition, a number of empirical studies have emerged, with the aim of discovering different expressions of “cussedness” in real life (e.g. anti-covid measure parties in public squares), but also in works of art and popular culture. Žikić’s contribution is in showing the existence of misanthropy in the functioning of certain social institutions and relations, public behavior and opinions, where it contradicts the proclaimed sociocultural values, and is despite this accepted as a legitimate sociocultural practice (Žikić 2015, 2016b, 2021b).

Another novel discipline at the Department, that of *Anthropology of emotions*, has been developed by Vladimira Ilić. The author treats emotions as cultural phenomena and approaches them from a constructivist perspective (Ilić 2014a). She explored their sociocultural aspects (as opposed to psychological ones), which means focusing on the ways in which they are imagined, shaped and transmitted through popular culture products (such as commercial films), on their use in political contexts (e.g. creating empathy in media campaigns for top political figures), and on the situations in which certain emotions are constructed (e.g. during the crisis caused by Covid-19 or due to changes brought about by migration).

So far Ilić’s research has been directed to the *study of different emotions, such as fear, happiness, despair, shame, as well as the states and capacities for empathy and nostalgia* (Ilić 2010, 2014b, c, 2019). The most attention was paid to fear. While studying American commercial films, the objects of inquiry were selected so as to treat different sources of danger that pose threat to individuals, groups or the whole humanity. Within the conceptual framework of the culture of fear, communicational approach was applied in the analysis of fears of ethnic and cultural others, foreigners and strangers, climate change and science, and fears of terrorism. From the study of imagined and produced notions of fear, the most recent research has moved to the exploration of this emotion among real people in real time, and in concrete situations. The contribution of this research is in bringing attention to the significance of studying sociocultural aspects of emotions – their construction in different contexts, as well as their social and cultural roles in different segments of social reality.

Nostalgy has also been explored by other authors, primarily from the point of view of *designing a precise theoretical and methodological framework for studying nostalgic narratives*, esp. those created around the previous “golden age” eras in Yugoslav history, known as “Yugonostalgia”. Within the interdisciplinary process, anthropologists and psychologists are to join forces in analyzing three kinds of nostalgia narratives – the life stories as folk narratives, personal memories as product of individual remembering, and oral histories (describing social, economic and other elements of the past) as the foremost form of nostalgia narrative (Antonijević et al.  2013; Kovačević et al. 2013).

*Anthropology of old age* is another subdiscipline related to human condition, which has been developed by Ljubica Milosavljević. Her work represents the first comprehensive study of old age as a social phenomenon, the topic which has previously been marginalized in national anthropology. The research primarily centers around construction of old age as a social problem in Serbia, and develops within a few fields. One field deals with the *state-organized homes for the elderly*, which exist within a wider range of institutions, from shelters for the poor to elderly homes. Another field deals with the *pension systems*, looking at the initial pensions established by the state in socialist times to the most recent private pension funds appearing in the time of neoliberal capitalism. The third field explores *“love in the third age”*, that is, partner relationships of the people accommodated in old people’s homes. Within the fourth field, Milosavljević analyses old age as a socio-cultural category, largely dependent on the overall social, political, and economic trends in Serbian society, but also on international influences that further shape it. Therefore, old age is not a fixed phenomenon, but an *expression of continuous social negotiations and adaptations to a whole range of diverse factors*. This approach thus separates the category of social age from that of age as a biological category (Milosavljević 2014a, b, 2018, 2019).

Other author’s studies encompass new related themes, such as political role of pensioners, limitations of the aging body, paradox of free time in old age, strategies of life style choices of retired guest workers, representation of old age in visual media, and old age as an exploratory resource for other sciences oriented towards the elderly (Milosavljević 2008, 2010, 2011).

Another subdiscipline related to human condition is *Anthropology of health and wellbeing*. Contribution to this subdiscipline has been made by Nevena Milanović, with focus on two fields, namely *Anthropology of drinking*, and *Anthropology of sports and fitness*. Within the first field, few themes have emerged, namely: cultural conceptualizations of drinking, spatial and gender aspects of drinking, the construction of drinking as a social problem, the “tavern culture”, as well as various drinking practices. The studies include a critical review of anthropological theories on alcohol consumption in the 20^th^ century (with focus on the theoretical and methodological turn of the 1960s), analysis of new “tavern culture” that explores correlation and interconnection between spatial behavior, music, identity and alcohol consumption treated as social categories, influenced by political and economic transformations in a society. The author’s main stance is that in order to understand all the dimensions of drinking as a social problem among young people, we should try to comprehend the meanings they attribute to drinking and drunkenness, which they often do not perceive as a personal and social problem, but portray it as acceptable behavior (Milanović 2018, 2019). Within the second field, related to sports and fitness, Milanović’s research deals with the themes of beauty ideals, fitness culture, as well as physicality and sports. The focus is on social conventions about physical appearance, whereby the body is perceived as a resource shaped by culture. The athletic, fit body is analyzed as a product of a specific discourse, in this case – fitness culture (Milanović 2020).

The *Anthropology of new medical (bio)technologies*, established as the new subdiscipline in Serbian anthropology by Zorica Ivanović, combines the study of the body, society and medical technologies. Themes developed through the research of this author comprise three interrelated areas. The first field concentrates on the body, while *exploring the body as a socio-cultural artifact*, and anthropological theories related to the issue of physicality. The second field examines *the body within society*, dealing with biopolitics and biosociality as well as with sexuality and identity issues. The third area deals with *medical biotechnologies* per se, while presenting up-to-date anthropological research of new (bio)technologies and exploring emerging relations between reproductive medical technologies and relevant public policies (Ivanović 2011, 2012, 2018).

*Anthropology of the (Covid-19) pandemic* is a subdiscipline that has emerged vividly in the midst of the global crisis caused by the Covid-19 pandemic. The Faculty of Philosophy launched the research project entitled “Man and Society in the Time of Crisis”, with the aim of looking at the implications of the current crisis on various aspects of people’s lives and on various spheres of social reality.

The project which started in 2020 and is continuing, involved 20 research teams and a total of more than 200 researchers, with a significant number of teams having an interdisciplinary character. The members of the Department have participated in the project, with papers in two edited volumes, *Covid-19 in Serbia ‘20* (Žikić 2021a), exclusively anthropological, and in *Everyday Life and Social Responses to Epidemic Crisis 1914-2020* (Ristović 2021), joined with historians. The themes dealt with in the first volume are: the absence of the social-humanistic approach in the state response to Covid-19 in Serbia in light of Lisbon Declarations on Humanities, avoidance and management of risk, crisis of pluralism and its consequences, quality of life of the elderly during the pandemic, understanding the pandemic in the post-truth era, practices of achieving physical and emotional well-being, contemporary legends, rumors and conspiracy theories about the Covid-19 pandemic, fear in the time of corona virus, modification of elements of the intangible cultural heritage, culprits and victims of the pandemic in public discourse, and understanding the epidemic through use of Foucault’s models of coping with contagious diseases. The anthropological themes dealt with in the second volume were: social and cultural practices of physical and social distancing during the epidemic, and adaptation of everyday life to changed circumstances through application of concepts of “resilience” and “resistance” to state measures.

Three authors intensely studied social and cultural consequences of the pandemic in a number of articles, which dealt with symbolic use of Covid-19 in Serbian public speech (Pišev et al. 2020), new cultural and social normality at the start of the pandemic (Žikić et al. 2020), communicative dissonance related to epidemiological danger announced by government officials, doctors and the national Crisis Committee during the epidemic in Serbia (Stajić et al. 2021), and vaccine refusal as an example of social solipsism, i.e., illusions about reality based on distrust of the dominant political, scientific and above all, medical discourses (Žikić 2022).

### Folklore and popular culture

*Anthropology of folklore* has had a lengthy history at the Department. The fields and themes developed along the way by different authors have had individual pathways, but have also come to intertwine and cross each other. Even though primarily applying theoretical and methodological frameworks of the folklore studies, their work may be characterized as simultaneously belonging to the subdisciplines of economic and political anthropology, anthropology of transition, urban anthropology, and anthropology of contemporality.

The early works of Ivan Kovačević on myth and ritual originating from mid-1980s, set forth the initial basis of modern folklore studies at the Department. The three volumes of *Semiology of Myth and Ritual* (Kovačević 2001b) are compilations of the earlier work, which include methodological and interpretative approaches, but also a number of texts analyzing phenomena of contemporary urban and rural as well as of political and economic folklore in Serbia. Myths and rituals are here treated as modes of communication with the aim of uncovering their hidden meanings and functions. Kovačević’s more recent research has produced a number of thematically profiled monographs. *Anthropology of Transition* (Kovačević 2007), comprises two parts -- folklore of transition and anthro(po)political economics, both dealing with *the social phenomena marked by the previous two decades of transition from socialism to capitalism in Serbia*. Using structuralist approach, he first analyzes legends about games of chance (betting), i.e., gain of unexpected wealth, investment into business and disappointment in the newly installed (capitalist) credit system, as well as about success, the self-made man and a lost fortune. In the second part, he treats the issue of public debates on placement and removal of kiosks from the streets (which were at the time a source of cheap and easily accessible goods) from two central Belgrade communities, based on the critique related to esthetics, industrial design and urbanism as well as social and economic policies. The author also considers gastronomic festivals that combine traditional knowledge of preparing food and doing business with modern technologies, organized throughout Serbia with varied popularity.

Another book by the same author, *Urban Legends: Essays on American Folklore* (Kovačević 2009b), is based on the argument that the development of information technologies, has recently made feasible the previously unimaginable reciprocity in the field of anthropological research. While only the wealthy scientific communities had been partaking in global research, and the rest of the world was deprived of this possibility, the fast flow if information has made possible that each of its users be in the possession of relevant information for the study of, until now, unapproachable turfs, such as the culture of the USA. Urban legends epitomize a paramount illustration of the possibility of examining American folklore from any world point, not least Serbia. This is possible, for urban legends generated in America are disseminated through the standard channels of globalization and through the World Wide Web. The legends analyzed deal with themes of destroyed car out of husband’s jealousy, a luxury car bought for a low price, a woman paying high amount of money for the recipe of a cake she spotted at an expensive restaurant, a young couple avoiding death and escaping a maniac, and Joe Magarac (Joe the Donkey), the mythical hero of American steel industry.

Monograph entitled *Transitional legends and Panics* (Kovačević 2011) continues with folklore analysis within the framework of *symbolic interactionism and interpretation of mass hysterias and urban legends*. Themes represented in the book deal with the problems brought by transition to capitalism in Serbia. Among them is a story of an old shoemaker who was not acquainted with the newly introduced credit payment and thus experienced financial collapse, and another about the success of restaurants opened by a Chinese and a Roma owner despite the competitive local environment fused with xenophobic attitudes. Tracing their origins, the author finds it mostly in the much older American legends that have with time spread via different media, and appeared in transformed versions in Serbia.

Initially exploring family relations in Serbian fairy tales based on Vladimir Propp’s semantics, and then researching folklore centered on the leaders of Serian revolution (i.e. Karadjordje Petrović and Miloš Obrenović, as leaders of the two major uprisings against the Ottoman rule, 2007a, b), Dragana Antonijević continued to deeply engage and modernize the study of folklore in Serbia. Her overall field of interest covers theory of folkloristics, anthropological approach to folklore, verbal oral prose genres, such as fairy tales, legends, anecdotes, myths, but also personal life stories, oral histories, and political folklore.

In her book *Essays in Anthropology and Semiotics of Folklore*, Antonijević (2010) sought to examine – from the transdisciplinary perspective characteristic of folkloristics as an eclectic discipline – several *innovative methodological proposals* which can contribute to the further study and interpretation of folklore. The essays draw from various disciplines – anthropology and structural anthropology, semiotics, narrative theory and sociology. Their common thread is the text as the main subject of study, or as the end product of complex folklore communication. The central chapters deal with the same basic methodology – structuralism, although applied in different ways and inspired by different structuralist scholars (Propp, Lévi-Strauss, Piaget, Greimas), and applied by the Serbian folklorist-philologist Nada Milošević-Đorđević, the American folklorist and psychologist Beverly Crane, and the author herself.

Deconstruction of urban legends that tell of *contemporary social problems related to children* has been of interest to Mladen Stajić. Dealing with the controversial media discussion on the theft of babies in maternity hospitals in Serbia (at the start of the 21^st^ c.), his monograph meticulously reconstructs the chronology of events within a political and social context, showing the mechanisms of social construction of a contemporary fairy tale (Stajić 2013).

Ethnographic research of digital folklore has appeared as the new trend in the Department. Within this field, Ana Banić Grubišoć and Dragana Antonijević analyzed the content, meanings and functions of contemporary folkloric creativity – *the internet humor* (Antonijević and Banić Grubišić 2013, 2021). The subject of analysis was digital visual-textual materials, most often expressed through the genre of internet mimes or the so-called Photoshoplore. Relying on approaches developed by American folklorists of the younger generation (Trevor Blank, Andrew Peck, Adrea Kita, etc.), an effort was made to perceive the local specificities and meanings of these globally popular internet genres.

With the increasing focus of Serbian anthropology on modernity, the popular culture as an unavoidable part of contemporary life, became a regular and important subject of anthropological inquiry since the last quarter of the 20^th^ century (Kovačević 2017a).

The 2010s have been characterized by a proliferation of fields of the *Anthropology of popular culture*, which is especially visible in the textual production in scientific journals, edited volumes, and monographs. In line with this, the edited volumes of the New Serbian Anthropology series (2013) are comprised of articles on music, commercials, film, TV shows, and science fiction. The journal *Issues in Ethnology and Anthropology* has also published thematic issues on popular culture, horror, music, literature, film, TV series, and commercials.

The consideration of *feature films* became a fixture in the anthropology of popular culture. Starting with Kulenović’s monograph *Social Ontology in the Film Avatar* (Kulenović 2011), the exploration of film has been pursued by a number of Department members to become one of the most bourgeoning fields up till the present.

As an illustration of a wide variety of film genres and themes considered, as well as theoretical and methodological approaches applied, the texts comprising the two thematic issues devoted to *Anthropology of film* (Kovačević 2017b) are here presented in more detail. The first volume was devoted to foreign, and the second to domestic (Serbian) films.

While analyzing the film *American Gangster* (2007), directed by Ridley Scott, Antonijević deals with the mythologization of gangsters through folklorization of the main character, Frank Lucas. The contextual analysis is dedicated to the relationship between criminal activity, competition, aggression, and the “American dream” as an ideal and a way of life, but also to the issue of ethnic and especially black criminal activity in American ghettos.

The text by Gačanović compares the anthropological and cinematographic understanding and representation of the urban poor. Theoretical and practical achievements of Oscar Lewis’s concept of the “culture of poverty” are compared with the representation of the poor in Vittorio De Sica’s film *Miracle in Milan* (1951). The aim of this comparison is to confront two viewpoints – one which aims to get to the scientific truth about poverty and the other – which gives a subjective artistic interpretation of the “old and romantic story about the rich man and the pauper”.

Using as an example Todd Solanz’s movie *Happiness* (1998), Ilić deals with cultural ideas on which (de)construction of a happy American family and happy individuals who “have everything” is based. It analyzes the director’s reflexive imagining of every character in the film, whose appearance is based on cultural attitudes and values of individuals, and which arises as an obstacle for establishing intimacy with other people and achieving happiness.

Žikić analysis a pessimist overview of the nature of human existence as presented in Michael Crichton’s science fiction movie *Westworld* (1973). Approaching it from the perspective of misanthropology, the author deconstructs the main message of the film, which pertains to the inexistence of a solution to the instability of the relationship between the normative order and human behavior, set up in a theme park where the androids serve human visitors in a variety of ways.

While choosing the science fiction film *Men and Chicken,* directed by Andres Thomas Jensen (2015), Kulenović aimed at shedding light on the hopes, fears and conceptual dilemmas caused by the advance of biotechnologies. By using a product of popular culture close to the experience of the general public, she analyzed the way in which the *imaginarium* of the film deals with the “paradox at the heart” of Western thought – the simultaneous claim that humans are animals and that animality is the polar opposite of what we hold to be human. Thanks to the increase of scientific knowledge and the development of technologies such as biotechnology, nanotechnology, bionics, robotics, artificial intelligence and virtual reality, humanity has entered an age marked by another “post” prefix – age of post-humanity, in which attempts are made to clearly dichotomize reality into humans and animals, humans and machines, natural and artificial, or real and unreal, thus giving more and more trouble to the categorical apparatus of culture.

Stajić analyses *Gattaca* (1997), a science fiction film, set in the “not-so-distant-future” in which genetic engineering of children has become a part of everyday life. This Andrew Niccol’s film is a dystopian vision of the world in which eugenics represents established social norm and stimulated practice, while biological conception carries a stigma of aberrant and irrational. While putting focus on religious symbolism in the film, Stajić discusses the argumentation for the idea that gene modification means espousing the role of the Creator, and seeks answers to the question of what it means to be human in a genetically deterministic world.

The second volume devoted to anthropology of film featured analysis of domestic films (Kovačević 2017c). Kovačević chose to analyze the film *Montevideo, Taste of a Dream* (directed by Dragan Bjelogrlić, 2010), one of the most successful blockbusters recently produced in Serbia, which tells stories about preparations for the qualifications of the Serbian football team in the First World Cup, held in Uruguay in 1930. The author finds the causes for film’s high popularity in the wide spectrum of its ideological content, evident in the oppositional relations established between the central characters of the film. The main oppositions are between the Yugoslav ideology of the state and the desire for an independent Serbia, between the bourgeois-patriarchal and free-celibate understanding of love and marriage, and hedonism as opposed to political activism. Local-patriotism, a bourgeois view of love and marriage based on romance, the idea of socially responsible entrepreneurship and support for football, are other oppositions that make up a wide array of ideological content offered by the film.

Basing her viewpoint on Miller’s conceptualization that film can be seen as belonging to the category of artistic artifacts of complex and long-term production, which implies multidisciplinarity and teamwork, Erdei analyzes Srdjan Karanović’s film *Something in Between* (1982). Action is situated between New York, Belgrade and Istanbul, and focused on a drama involving a “love triangle” between an American woman and two Serbian men, in Yugoslav times when the country was situated between the capitalist West and real-socialist East. Both explicitly and implicitly, the film deals with the “state of being in between”, considering historical continuity of such a state which produced certain mentality and self-identification, as well as the direction in which the Serbian society is moving after Tito’s death in the wider context of modernity in Europe.

While taking up the film *Enclave* (by Goran Radovanović, 2015), Slobodan Naumović uncovers the logic of a particular Serbian “contemporary historical film” and its presentation of the complex cohabitation of Serbian and Albanian children in enclaves in Kosovo and Metohija, the southern Serbian autonomous province administered by the international peace forces. Inspired by ideas of Cauton and Augé, Naumović argues that the contents initiated by the films remain at the same time irreducibly personal, shaped by the viewers’ unique characteristics and life histories. Precisely the complex relationship that is established in each individual case between the film as an active principle and the viewer as an active participant in the process of experiencing what is seen, and the subsequent production of meaning based on what was previously seen, represents a desirable field for anthropological research.

The anthropological analysis of the TV movie *Stairless* (by Marko Novaković, 2014) is taken up by Milosavljević due to her long-term research focus on the theme of old age. The analysis shows how the way in which the illness of the retired professor of neuropsychiatry – Alzheimer’s dementia – is presented, reflects all the problems faced by those who fall ill as well as their caretakers. The analysis further shows how the film ending (when due to lack of other possibilities, the protagonist is placed in a home for the elderly), brings the film viewers in a position to face the fear of old age, but also to deal with dilemmas and norms which regulate the handling of this issue in contemporary Serbian society.

The subject of Marija Brujić’s paper is a Serbian feature film *Dear Video* (by Goran Gajić, 1991). Two brothers with their families, one living in Germany as a well-established migrant worker, and the other living in their home village in Serbia, are communicating through VHS video tapes. Instead of facilitating their communication, the VHS video tapes actually reveal all that was suppressed and hidden from surface relationships, such as dishonesty, jealousy, and deceptions. The film shows discord between the two brothers, among cousins, villagers and different nationalities in Yugoslavia. Aim of the paper is to examine how this film deals with the issue of unsuccessful (socio-cultural) modernization of Serbian villages, in other words, why migrant workers did not have greater and more important influence on modernization of their places of origins.

Serbian film *Ring a Ring o’ Roses* (by Djordje Milosavljević, 2002) became the subject matter of Banić-Grubišić’s inquiry owing to its dealings with the ideas and cultural notions about ethnology and anthropology in Serbia. The action of the comedy is set up in a village at the time of the celebration of a family feast and a community ritual aimed at inviting rain, where a guest – pretending to be an ethnologist – is treated as an expert for authentic cultural traditions. Aside from the analysis of the representation of ethnology as a profession in this film, the paper considers the image of a fictional ethnologist/anthropologist in popular imagination. It also underlines the similarities and differences between domestic ideas about ethnology/ethnologists and ideas about anthropology/anthropologists in global pop culture, primarily in film and literature. Finally, the paper discusses the public image of the discipline by comparing the imaginary ethnologist/ anthropologist and the way in which real ethnologists/anthropologists work.

Recently, two monographs appeared dealing with analysis of *films and TV series focused on Yugoslav socialism*. Kovačević’s book *Film, Football and Politics* (Kovačević 2020), comprises texts that illustrate searching for the main socio-cultural themes embedded in the film through the analysis of meta ethnography and reception of the film in the society. Overall, Kovačević’s opus is a collection of interpretations of various real or narrated public “events” represented in films, in which he uncovers the aspects that are vague, contradictory, and contrary to what is possible. As an illustration, the text “Comrade vice president – Center forward, 1960”, puts a football match in the context of annual celebration of the founding of the village agricultural cooperative in late 1950s. The analysis uncovers elements of modernization processes as part of socialist development in a small Serbian town as well as the relationship of the common people to art and artists (participating in the decoration of the main cooperative hall) in this period. The ideological message of the film is that socialist transformation of the society leads to its modernization.

In the book *Modern Life in Prime Time* (2020), Erdei focuses on the anthropological analysis of a popular TV series, called *Theatre at Home*, shown in the 1970s and 80s on Yugoslav television. The author sheds light on the ways in which television – as technology, institution, medium and artefact, influenced modernization of post-WW2 Yugoslav society and its increasing links with the currents of Western modernity. Television is here viewed as an inseparable part of important social processes, such as modernization, urbanization and nation-building in the 20^th^ century. Television is thus, from the anthropological perspective, viewed as one of the (new) social and cultural institutions, as a form of contemporary social life, along with law, economics, art, religion, family and kinship relations.

### Arts: visual, literary, and musical

Art is a diverse range of human activity and its resulting products that involves creative or imaginative talent expressive of technical proficiency, beauty, emotional power, or conceptual ideas. Contemporary definition of the arts includes painting, sculpture, architecture, theatre, dance and other performing arts, music, literature, photography, film, and other media, such as interactive media related to digital computer-based systems.

Among the subdisciplines that belong to the broad family of Anthropology of the arts, it may be observed that quite a few have been developed by the Department members, especially since the 2000s. However, the initial exploration within this area may be recognized in the work of Slobodan Naumović, who conceptualized the subdiscipline of *Visual anthropology*. After early interest in uses of visuals in teaching ethnology in late 1980s, and film-making projects in late 1990s, his research since 2009 developed with focus in the fields of feature film, ethnographic film and photography.

It should be noted that there are sometimes different understandings about naming and dividing turf between Visual anthropology and Anthropology of film. Slobodan Naumović, sees these two areas as part of a whole. He thus opposes the view that Visual anthropology deals with documentaries and Anthropology of film with feature films. Naumović approaches both genres in the same way, the most important reason being that in the documentary film there is also plenty of staged content, i.e. the content premeditated by the director as well as by the actors themselves, which is in accordance with how they want to present themselves to the public. The film is here treated as a part of reality which it reflects, and which an anthropologist can extract with his analysis. Therefore, he finds the meaning of life in the film, sees its content not only as a value in itself, but also how it acts upon the viewers, including himself.^7^

Intellectual and methodological preoccupations of Naumović’s work related to exploration of the *feature films* were to set the criteria for determining national cinematography, approach the film from the philosophical and phenomenological perspectives, analyze the place of a film in the author’s overall poetics, as well as to produce an autoethnography of film perception.

The first theme focuses on the logic of self-presentation and self-understanding in the Yugoslav and Serbian cinematography (Naumović 2010). Application of Herzfeld’s concept of "cultural intimacy" on the analysis of Serbian films of different periods and genres aimed at deeper understanding of the production of auto-stereotypes in Serbian cinematography. This is the first domestic attempt to systematize the levels of manifestations of cultural intimacy in film (diegetic level - among the characters themselves, as well as the stories the characters tell; extradiegetic 1 - between the author of the film and the characters; extradiegetic 2 - between the domestic audience and the characters).

The second theme deals with contemporary Serbian historical film and its presentation of the complex cohabitation of Serbs and Albanians in enclaves in Kosovo and Metohija, the southern Serbian autonomous province administered by the UN peace forces (Naumović 2017). This is the first attempt at autoethnography of feature film reception, and the first attempt to constitute a comprehensive author’s poetics. The study aims to understand the director’s actions through which the logic of a particular film that deals with “history which is still ongoing”, practically influences the viewers of the film. It attempts a precise uncovering of the way in which audio-visual story of the painstaking rise of a friendship between an Albanian and a Serbian boy in an enclave in Kosovo and Metohija struggles with, resists, but simultaneously blends with the knowledge, expectations and memories carried by those who had the opportunity to view the film, including, foremost, the author of the study. The key question addressed is how, or rather, why certain films become meaningful in the lives of those who watched them.

While exploring *documentary films* as a researcher, Naumović’s theoretical and methodological inspiration was based on constructing comparative chronologies, application of Nichols’s systematization of author’s approaches in the analysis of individual author’s poetics, as well as shared anthropology. This was an attempt to, by using film, tell (verbally) and show (pictorially) theoretically relevant insights.

The themes pervading his work included history of ethnographic film, the relationship between ethnographic film and visual anthropology, ethnographic and documentary film festivals, and comparative analysis of two documentary film festivals (Naumović 2020a, 2021). A long-term project realized through documentary filming focused on intangible cultural heritage or Romanians and Serbs in Banat region, historical processes of cultural contacts and cooperation, and invention of tradition. Through film, he also dealt with the application of theatre therapy in overcoming social barriers faced by persons with disabilities. In addition, special works devoted to individual authors, dealt with wider themes, such as the reconstruction of traditional culture in ethnographic film (Balikci) and television reportage as a visual expressive style (Katić). A special study of an indigenous documentary and feature film author (Pantelić) was devoted to indigenous Serbian filmmaking, as well as to new media and self-presentation strategies of marginalized social strata in Serbia (Naumović 2018, 2020b).

Theoretical and methodological exploration of the field of *photography* was devoted to historical periodization, analysis of the logic of the use of photography in different theoretical approaches, application of the phenomenological approach, shared anthropology approach, and photo elicitation method. Themes studied encompassed historical review of the development of an anthropological approach to photography in Serbia, a review of the work on the systematization, interpretation and digitization of the photo documentation of the French Society of Bor Mines (Naumović and Radivojević 2015), and analysis of the life-world of a street photographer and his way of experiencing and depicting urban spaces in Bor. One particular study looks at the industrial heritage of Bor as a key national resource, which may be protected if the arguments of industrial archaeology, ethnology of mining, political anthropology, and visual anthropology (within which a photo repositorium tracing a hundred years-long life of the mine has been created) is put into use against infiltration of transnational companies dealing with exploitation of local natural resources (Naumović 2013d).

*Literary anthropology and Anthropology of literature* are novel subdisciplines in Serbian anthropology, that have been developed since the later 2000s. *Experimental ethnographic writing* appeared in Vučinić Nešković’s monograph *Christmas in the Bay of Kotor* (2008), where she explored writing in different genres to accommodate the ethnographic data collected on family, collective, and church Christmas festivities in Montenegro. Among four chapters that constitute the heart of the book, two were written as ethnographic diary, one as an ethnographic interview transcript with commentaries, and the last one as a theatre scenario. The last one, dealing with the collective youth initiative of burning yule logs by the local Serbian Orthodox church that occurred in 2004 at the Luštica Peninsula, describes the preparations and the festivity itself, based on the authentic video and audio material made by the author.

In the latter part of 2010s, Gordana Gorunović has been systematically developing this subdiscipline. Her general approach is interdisciplinary, whereas theoretical concepts, analytical methods and models of text interpretation are taken from literary anthropology and anthropology of literature, social and cultural anthropology, social phenomenology, historiography, literary theory and criticism, and cultural studies.

The author opens a new field in national ethno/anthropology, trying to *thematize the relationship between anthropology and (national and world) literature*. Earlier texts, recently collected in the book *Essays in Ethnography and Anthropology of Literature* (Gorunović 2022) are diverse in terms of themes they deal with – from the literary and documentary work of writer (and communist cadre) Mihailo Lalić and his connection with the ethnographic strategy of Serbian bourgeois ethnology, the dystopian novel *The Possibility of an Island* by the contemporary French writer Michel Houellebecq, the autothanatography (account of one’s death experience) of the American anthropologist Robert F. Murphy’s *The Body Silent*, fictional and documentary writing by B. Vongr (Sreten Božić) in Australian literature, to classic Native American autobiographies such as Radin’s popular work *Crashing Thunder* and Kroeber’s biographical-historical book about the Indian Ishi. The last essay is devoted to the ethnobiography of a village woman from Montenegro, viewed from the perspective of Schutz’s social phenomenology. The texts consistently follow the same direction of interest – reading literature through anthropological eyes, as well as, conversely, interpreting ethnographic and anthropological theoretical writing as a literary creation. Beside the described cases, and the contextual circumstances of their origin and development, the reader is offered information about the reception and disputes they caused.

Within the wider domain of Anthropology of the arts, Marija Ajduk (Ristivojević) has been developing the subdiscipline of *Anthropology of music* in domestic terrain since 2010. Music as the subject of research in national science until then was mostly analyzed from the perspectives of ethnomusicology, musicology, sociology, and history, while the anthropological interpretation of music as a sociocultural phenomenon was insufficiently developed, and therefore poorly visible in Serbian scientific community. With that in mind, the author’s goal was to establish a theoretical framework that would analyze music in interaction with the cultural and social processes that make up our everyday life.

Ajduk’s studies are based on the idea that music is not only a product of the local cultural environment, but that with its many meanings, it influences the construction of local identities (Ristivojević 2012; Ajduk 2014, 2018a). In this sense, the accent is placed in the field within which *correlation between music as a concept and the idea of local identity* is explored. The “new wave” in rock and roll music, which marked the 1980s, was a global phenomenon that originated in the large urban centers of the USA and Great Britain. However, the focus of author’s research was on the local variant of the new wave that existed in former Yugoslavia. Relying of Cohen’s theoretical stance that the identity of a place can be partly built with the help of music as a signifying element (e.g. concert venues, rehearsals spaces, popular meeting places of like-minded music lovers, etc.) and Regev’s concept of authenticity in rock and roll music, Ajduk produced a study of the influence of the new wave on the local identity in Belgrade.

Besides the earlier focus in the field of music and local identity, further research of the author explored other fields within the same subdiscipline. One field, continues to focus on new wave, and examines its presentation in popular culture media, such as in TV series and documentary film, treating it as a social movement in socialist Yugoslavia. Most recently, a book analyses presentation of Yugoslav new wave in the 1979-1985 period press (Ajduk 2021). Another field incorporates the study of the traditional and modern aspects of music, expressed in genres such as “ethno-punk and “world music” (Ristivojević 2014; Ajduk 2018b).

Later, when the Department set on the development of studies of popular culture, a large publishing project on this subdiscipline encompassed Anthropology of music. A thematic issue comprising three volumes, edited by Mladen Stajić, and published in the 2018-2019 period, included 20 articles, out of which most were written by the Department members or graduates. Similar to Ajduk’s stance, music here represents an important aspect of identity of both individuals and groups and an inevitable part of everyday life impregnated with cultural codes that are a part of the symbolic system of our society. Consequently, a number of anthropological topics that can be studied through music, such as identity, gender, ethnicity, religion, globalization, urbanization, migration, economics, markets, popular culture, symbols, performance, dressing, etc. were explored (Stajić 2018).

## Conclusion

The overview of the existing subdisciplines, fields and themes that have been developed at the Department of Ethnology and Anthropology since 2006, as found in the academic publications of the faculty members, indicates two main trends, namely a proliferation of a wide range of research interests, and an accelerated dynamic of publishing.

Both of these trends came out of the Department politics of stimulating research in various directions. Research fields that had until then been unexplored were picked up and developed by the senior faculty, who in turn encouraged the junior faculty to undertake novel research topics for their doctoral theses. In addition, stimulating team work, through publication of coauthored texts and compilation of thematic volumes, enhanced both the homogenization of the discipline and its strengthening, but also development of its diversity and individual creativity.

The overview indicates a wide array of theoretical and methodological approaches as well as types of analyses that were applied to ethnographic data, gathered from a variety of sources. The wider (macro) theoretical approaches include diffusionist, communicational, feminist, folklorist, literary-historical, literary-anthropological, materialist, neo-functionalist, phenomenological, semiotic, social constructivist, shared anthropology, socio-cultural ontology, structuralist, and symbolic approaches. In addition, within individual subdisciplines and fields, more specific (mezzo) theories have been applied. For example, economic anthropology research has been informed by theories of economic and organizational cultures, state administration, neoliberal economics and state, theories of consumption, etc. While studying specific themes, authors used different types of analyses, such as: content, discourse, narrative analysis; formal, functional, structural analyses, as well as socio-spatial, comparative, cognitive, and a variety of others. The ethnographic data acquired through research was mainly collected through qualitative anthropological methods, but in some cases also in combination with quantitative methods.

The main contribution of the research results presented in published works of the faculty is reflected in the analytical consideration and interpretation of contemporary socio-cultural phenomena and processes, which have not been the subject of more extensive research in domestic ethnology and anthropology. Application of the new theoretical-methodological apparatuses in this research also contributed to the scientific explanation of often studied but so far insufficiently explained phenomena. Another contribution is in that the study of socio-cultural phenomena has often been done within the framework of globalization processes and their manifestations at the national and local levels. The previous aspects of research enabled the systematization of knowledge about certain anthropological phenomena as well as creation of different kinds of typologies, both abstract and specific. Altogether, it may be concluded that the research performed within the Department of Ethnology and Anthropology shows originality, not only within national ethnology and anthropology, but also within the framework of world anthropologies.

One of the main results of this study is the fascinating fact that an institution in a small country such as Serbia has developed such a rich and dynamic discipline. It is especially worth noting that such richness of the discipline has been developed in circumstances in which institutional funding for fieldwork has been lacking. This crisis in turn stimulated search for other “field trips”, such as into the domains of visual, literary, and music arts, and varied popular culture media. Another way to deal with crisis was to rethink the status of the discipline, while examining its intellectual history, epistemological and methodological grounds, as well as the scientific and educational system it found itself in at present.

And one last observation. Only while rounding up this text, I realized that it not only presents a mosaic of the research themes, fields and subdisciplines of ethnology and anthropology, but also of history and transformation of Serbia (within and outside Yugoslavia), and the major issues the country and its people face today. In fact, the research themes cover a very long period and different political and economic eras, starting from the time of monarchy, through socialism, and the entry to neoliberal capitalism (often called postsocialism). The “transitional period” between the latter eras had an especially important place in the research of the Department.

## Data Availability

The data used and analyzed in the study are available at the Faculty of Philosophy, University of Belgrade website (https://www.f.bg.ac.rs/etnologija_antropologija/zaposleni_od), as well as from the author on reasonable request.

## Abbreviations

DEA: Odeljenje za etnologiju i antropologiju Filozofskog fakulteta, Unierziteta u Beogradu (Department of Ethnology and Anthropology of the Faculty of Philosophy, University of Belgrade)
DS: Dosije Studio
IEA: Etnoantropološki problemi (Journal: Issues in Ethnology and Anthropology)
SGC: Srpski genealoški centar (Serbian Genealogical Center)

## Footnotes

1 The journal *Issues in Ethnology and Anthropology* was founded in 1987 and published until 1998, to be relaunched in 2006, thus in the same year when a new journal, *Anthropology* was founded.

2 Note: This overview includes the present members of the Department of Ethnology and Anthropology, i.e., those that were employed at the Faculty of Philosophy at the time this article was being written, which is the summer and fall of 2022.

3 Anthropology of museums and museology has been developed mainly by externally engaged (or part-time employed) staff, previously Ljiljana Gavrilović, from the Ethnographic Institute, and presently Marina Simić, from the Faculty of Political Sciences.

4 Somewhat later, Serbian ethnology/anthropology dealt mostly with the problems of older migrations, especially those across the Atlantic (so called pečalba), as well as of Serbian Diaspora, and questions of the status and identity of Serbian national minority in the neighboring countries.

5 In his major monograph, Naumović shows that by effectively using tradition as a key symbolic value in nationalist and populist rhetoric, as well as in spectacular political rituals, in Serbia of the late 1980s and 1990s, the crisis of political legitimacy of the collapsing political system was temporarily resolved and the system was changed without a change in power holders.

6 Тhe review of the development of Economic Anthropology in Serbia relies mainly on Erdei’s text, published in Newsletter of Anthropology of Economy Network of EASA 2021, no. 1 (March 2021), which appeared in both short and long versions. The long version may be found at https://drive.google.com/file/d/1vcS_Bgy2iAy7u1aZqXbloNYzAF_deWWw/view.

7 Most of the other Department members, as shown in the previous section on anthropology of the feature film, as part of Anthropology of popular culture, treat the film like any other text, to be read within a specific socio-cultural context. In this sense, they see film as a medium through which phenomena portrayed can be read out in other media as well, i.e. as a channel (medium) through which meanings are transmitted.
